# Dysfunctional telomeres through mitostress‐induced cGAS/STING activation to aggravate immune senescence and viral pneumonia

**DOI:** 10.1111/acel.13594

**Published:** 2022-03-21

**Authors:** Nianyin Lv, Yufang Zhao, Xiaoyi Liu, Lusha Ye, Zihao Liang, Yanhua Kang, Yeping Dong, Wei Wang, Narasaiah Kolliputi, Liyun Shi

**Affiliations:** ^1^ 118211 Department of Immunology and Medical Microbiology Nanjing University of Chinese Medicine Nanjing Jiangsu China; ^2^ 74653 Department of Basic Medicine Jiangxi Medical College Nanchang Jiangxi China; ^3^ 66478 Department of Pharmacology and Physiology University of Rochester School of Medicine and Dentistry Rochester New York USA; ^4^ 118211 Institute of Translational Medicine Zhejiang Shuren University Hangzhou Zhejiang China; ^5^ Department of Clinical Laboratory the Tongde Hospital Affiliated to Zhejiang TCM University Hangzhou Zhejiang China; ^6^ 7831 Division of Allergy and Immunology Department of Internal Medicine Morsani College of Medicine University of South Florida Tampa Florida USA

**Keywords:** macrophage, respiratory virus infection, senescence, STING, telomere

## Abstract

Disproportionately high incidence and mortality of respiratory infection such as influenza A virus (IAV) and SARS‐CoV‐2 have been evidenced in the elderly, but the role and the mechanism of age‐associated immune deregulation in disease exacerbation are not well defined. Using a late generation of mice deficient in telomerase RNA (Terc^−/−^), we herein demonstrated that aged mice were exquisitely susceptible to respiratory viral infection, with excessive inflammation and increased mortality. Furthermore, we identified the cGAS/STING pathway, which was essentially induced by the leaked mitochondrial DNA, as a biologically relevant mechanism contributing to exaggerated inflammation in Terc^−/−^ mice following viral infection. Innate immune cells, mainly, macrophages with shortened telomeres, exhibited hallmarks of cellular senescence, mitochondrial distress, and aberrant activation of STING and NLRP3 inflammasome pathways, which predisposed mice to severe viral pneumonia during commonly mild infections. Application of STING inhibitor and, more importantly, senolytic agent, reduced the burden of stressed macrophages, improved mitochondrial integrity, and suppressed STING activation, thereby conferring the protection for Terc^−/−^ mice against respiratory infection. Together, the findings expand our understanding of innate immune senescence and reveal the potential of the senolytics as a promising treatment to alleviate the symptom of viral pneumonia, particularly for the older population.

## Graphical abstract

Together, we showed that senolytic agent conferred profound protection of Terc^‐/‐^ mice against respiratory primarily through reducing burdens of senescent cells, improving mitochondria integrity, and hence dampening STING‐mediated pathogenic inflammation.

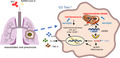

## 1 INTRODUCTION

Respiratory virus infection caused by devastating pathogens such as influenza A virus (IAV) or severe acute respiratory syndrome coronavirus 2 (SARS‐CoV‐2) affects people of all nations and ages, with a particular collapse in morbidity and mortality in older populations (Akbar & Gilroy, 2020). Further complications including secondary bacterial infections, severe pneumonia, respiratory failure, and even cardiovascular and musculoskeletal disorders are frequently evidenced in the elderly. The clinical observations indicate that aging is a critical risk factor for severe respiratory diseases and might be a potent target to be manipulated for better outcome (Ackermann et al., 2020). Although our current understanding about the age‐associated exacerbation in respiratory infection is preliminary, evidences have indicated that deteriorated immune systems during aging contribute substantially to this disorder (Kelley et al., 2020). Clinical data from severe IAV and COVID‐19 patients reveal that aberrantly activated innate immune cells, particularly monocyte‐macrophages, are critically implicated in the aggravated pathology in the elderly (Lee et al., 2021; Schultze & Aschenbrenner, 2021), which prompts us to further study how aging process impacts the innate immunity and disease progression.

The individual immune system is affected by and also influences the aging process. Age‐dependent decline in immune cell resilience and immunological competence, along with sustained release of inflammatory mediators that is termed as senescence‐associated secretory phenotype (SASP), constitute the hallmarks of immunosenescence (Xu et al., 2020). It has been demonstrated that aged lymphocytes are characterized by a gradual loss in naive cell pools and response plasticity. By contrast, innate immune cells, particularly macrophages from the elderly, tend to adopt activated phenotypes with abundant release of inflammatory mediators, causing widespread tissue injury upon commonly non‐lethal infections (Minhas et al., 2019). A successfully confined viral disease is featured with early production of antiviral effectors followed by lower level of the inflammatory response, whereas lethal pulmonary viral infection in the old population is frequently associated with exuberant inflammatory cell infiltration and delayed interferon generation (Arunachalam et al., 2020). Therefore, identifying the source of hyper inflammation is pivotal for our understanding of age‐associated exacerbation in respiratory viral infection.

During viral infection, the innate immunity and inflammatory response are initiated by sensing the pathogenic components via pattern‐recognition receptors (PRRs) such as toll‐like receptors (TLRs), retinoic acid‐inducible gene I (RIG‐I)‐like receptors (RLRs), and the cyclic GMP‐AMP synthase (cGAS)/stimulator of IFN gene (STING) axis (Wu & Chen, 2014). It is noted that in addition to virus‐derived molecules such as viral proteins, DNA, and RNA, innate immune cells also recognize and react to the “danger” signals that are released from stressed and senescent cells. With aging, cells undergo profound functional alteration involving replicative exhaustion, DNA damage, redox imbalance, and oncogenic activation, which may in turn trigger the release of damage‐associated molecular patterns (DAMPs) such as HMGB1, ATP, and mitochondrial DNA (mtDNA) to activate innate immune cells via PRRs (Tumburu et al., 2021). Among these changes, age‐related impairment in mitochondrial quality control is considered a key event contributing to exacerbated inflammation.

Mitochondria are known as a vital organelle in charge of a broad spectrum of cellular activities ranging from oxidative phosphorylation, ATP synthesis, autophagy, apoptosis to immune and inflammatory responses (Mills et al., 2017). Mitochondrial DNA (mtDNA) and the related bioactive molecules mediate these processes and are essential for maintaining cellular homeostasis and physiological activity. The fitness and integrity of mitochondria, however, would gradually deteriorate with aging. As a result, the mitochondria display diminished abundance, reduced membrane potential (MMP), compromised ATP‐production capacity, and elevated oxidative stress (Dou et al., 2017; Smith et al., 2020; Vizioli et al., 2020). In particular, mtDNA would be released from dysfunctional mitochondria and recognized by cellular surveillance system, primarily the cGAS/STING system, to incite the immune and inflammatory responses. cGAS is a cyclic DNA sensor to generate second messenger cyclic GMP‐AMP (cGAMP) for activating the key signaling adaptor STING, which subsequently initiates the NF‐κB and IRF3‐driven pathways to promote inflammatory cytokines and IFNβ production (Decout et al., 2021). Due to its critical role in connecting cellular intrinsic stress to pathogenic signals, the cGAS/STING axis has been implicated in aging and age‐associated inflammapathology (Yang et al., 2021). However, its relevance to exuberant inflammatory responses in the elderly, particularly during respiratory viral infection, is yet to be addressed.

Telomeres are repetitive DNA sequences occupying at the ends of chromosomes and play an essential role in maintaining genomic integrity and stability (Chakravarti et al., 2021). Dysfunctional telomeres due to excessive attrition or impaired telomerase activity lead to cellular replicative senescence, chromosomal instability, and ultimately organism aging (Sahin et al., 2011). Thus, defective telomere has been suggested as a driving force as well as a risk factor of aging‐related pathology. In support of this, recent data showed that adults with inherently short telomeres were prone to worsened inflammapathology and increased mortality during SARS‐CoV‐2 infection (Sanchez‐Vazquez et al., 2021). In mice, genetic deletion of telomerase RNA (Terc^−/−^) causes accelerated telomere shortening, premature aging, as well as age‐related immune deregulation and diseases (Blasco et al., 1997). For these reasons, we herein utilized the late generation of Terc^−/−^ mice to explore how the aging process impacted the innate immune response and disease manifestation during respiratory viral infection. The results showed that Terc^−/−^ mice were exquisitely vulnerable to IAV or SARS‐CoV‐2 infection, characterized by exacerbated lung inflammation and increased mortality. Mitochondrial distress‐induced cGAS/STING activation was identified as a significant mechanism underlying macrophage hyperactivation and exacerbated inflammapathology. Accordingly, the inhibition of the STING pathway or, more strikingly, elimination of senescent macrophages by senolytics alleviated pathogenic inflammation and increased hosts’ tolerance to respiratory viral infection. The findings provide a novel insight into the aging‐associated immunopathology and suggest a potential therapeutic strategy for respiratory viral disease, particularly for the elderly.

## 2 RESULTS

### 2.1 Dysfunctional telomeres render mice more vulnerable to respiratory viral infection

**FIGURE 1:**
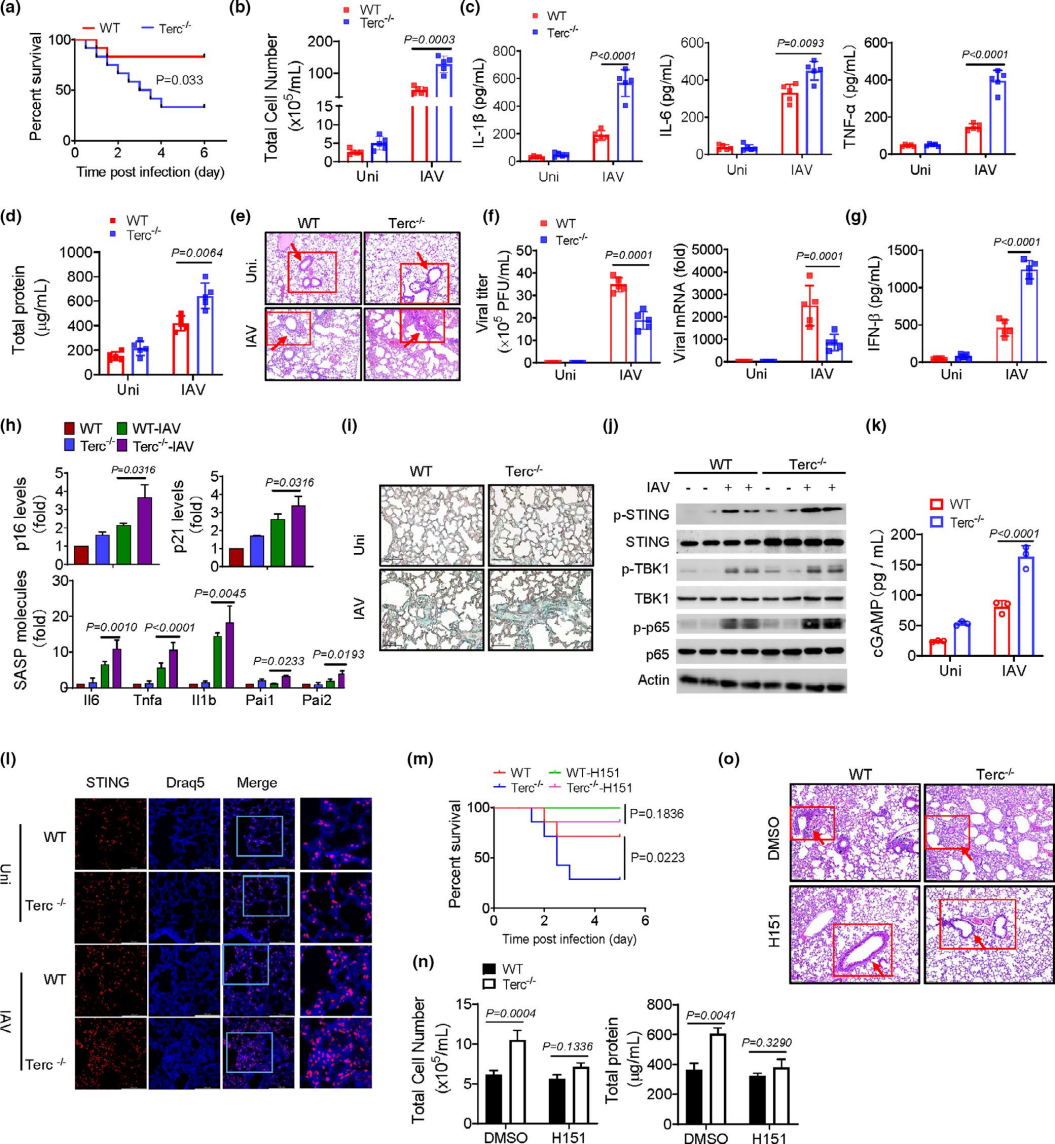
Dysfunctional telomeres increase mice vulnerability to respiratory viral infection through the cGAS/STING pathway. (a) WT and Terc ^−/−^ mice (*n *= 12) were intratracheally (i.t.) challenged with influenza virus A/PR8 strain (1 × 10^8^ pfu/mouse). Animals were monitored every 10 h for survival rate. The Kaplan–Meier and log‐rank methods were used to analyze the data. (b–g) WT and Terc ^−/−^ mice (*n* = 5) were infected with influenza A/PR8 strain (1 × 10^4^ pfu/mouse) or PBS (Uni) for 48 h, and then sacrificed for functional analysis. Counts of total cells in BALF (b); BALF levels of proinflammatory cytokines (c); Protein leakage in BALF (d); Representative H&E staining of lung tissues (e), sites of inflammatory infiltrate were marked; Quantification of viral titer and mRNA in lungs (f); BALF level of IFNβ (g). (h‐l) qPCR assay of p16, p21 and SASP‐related genes in lung tissues (h); Immunohistology staining of SA‐β‐Gal in lungs (i); Immunoblotting of STING and the related signaling molecules in lung tissues (j); BALF level of cGAMP (k); Confocal staining of DNA dye Draq5 (cyan) and STING (red) (l). (m) WT and Terc ^−/−^ mice (*n *= 7) were pretreated with H151 (7 mg/kg) or DMSO for 3 days, followed by A/PR8 infection. Animals were monitored every 10 h for survival rate. (n, o) WT and Terc ^−/−^ mice (*n *= 3) were pretreated with H151 or DMSO for 3 days, followed by A/PR8 infection for 48 h. Counts of total cells and protein leakage (n) in BALF; Representative H&E staining of lung tissues (o). Shown are representative images, Bars, 100 μm. The data from one of three independent experiments are expressed as means ± SD

To elucidate the role of age‐associated immune deregulation in host response to respiratory virus infection, we established an infection model with influenza A virus (IAV) using the 3^rd^ generation of Terc^−/−^ mice (G3), a prototypic animal model characterized by premature aging and dysfunctional telomeres (Blasco et al., 1997). The result showed that compared with wild‐type (WT) mice, Terc^−/−^ mice exhibited a significant decrease in survival rate when challenged with a lethal dose of IAV strain (Figure 1a). In parallel, influenza infection caused increased infiltration of total cells and proinflammatory cells such as macrophages and neutrophils in Terc^−/−^ mice relative to WT animals (Figure 1b and Figure S1a‐b). The production of inflammatory cytokines IL‐6, TNFα, and especially IL‐1β, an effector molecule induced upon inflammasome activation, was augmented in Terc^−/−^ mice following IAV exposure (Figure 1c, Figure S1c‐d). The amount of protein leaked in bronchoalveolar lavage fluid (BALF), indicative of lung permeability and injury, was increased in mice with defective telomeres (Figure 1d). Histopathologic analysis consistently revealed exaggerated pulmonary inflammation and injury in Terc^−/−^ mice (Figure 1e). Despite aggravated tissue damage, Terc^−/−^ mice demonstrated a lower level of viral loads in both BALF and lung tissues (Figure 1f). Associated with this, the production of IFNβ, an essential antiviral effector factor, was enhanced in Terc^−/−^ mice relative to their WT littermates following IAV exposure (Figure 1g). The data thus indicated that the innate antiviral immunity was not significantly impaired in mice with shortened telomeres.

To further substantiate the telomere‐related disease exacerbation, we also infected mice with vesicular stomatitis virus (VSV), another kind of respiratory RNA virus. Similarly, exacerbated pulmonary inflammation and injury was induced in Terc^−/−^ relative to WT mice upon infection (Figure S2a‐d). Together, our data indicated that mice with dysfunctional telomeres were increasingly vulnerable to respiratory viral infection, with exaggerated lung inflammation and injury and hence increased lethality.

### 2.2 Terc^−/−^ mice display senescent signatures and aberrant STING activation underpinning aggravated viral pneumonia

Defective telomeres have been proposed as a hallmark of cellular senescence, which may cooperate with senescence‐associated secretory phenotype (SASP) to predispose hosts to hyper‐reaction to normally non‐lethal infection. Indeed, Terc^−/−^ mice displayed a higher level of senescence signature genes p16 and p21 (Liu et al., 2019) compared with their WT controls. In addition, they expressed increased amounts of SASP‐related inflammatory molecules including IL‐1β, IL‐6, TNF‐α, and plasminogen activator inhibitor (PAI)‐1 and 2, particularly following IAV exposure (Figure 1h). Lung tissues in Terc^−/−^ mice exhibited enhanced staining of β‐galactosidase (β‐Gal) (Figure 1i), a well‐known characteristic of senescent cells (Debacq‐Chainiaux et al., 2009). The results thus indicated that dysfunctional telomeres induced the senescent features in mice particularly following viral infection.

Cellular senescence is generally accompanied by stressful events such as aberrant release of mitochondrial DNA (mtDNA), which would induce the cGAS/STING pathway to drive the innate immune and inflammatory responses. Our data demonstrated that the phosphorylation of STING and the downstream signaling molecules including p65/NF‐κB and TANK‐binding kinase 1 (TBK1), the factors essential for the expression of the inflammatory cytokines and IFNβ, respectively, were enhanced in lung tissues or macrophages from Terc^−/−^ mice (Figure 1j and Figure S3a). The expression of type I IFN‐stimulated genes (ISG) was consistently boosted in Terc^−/−^ mice (Figure S3b). More supportively, BALF level of cGAMP, a specific cyclic dinucleotide driving STING activation, was increased in Terc^−/−^ mice relative to WT animals following IAV exposure (Figure 1k). Interestingly, we noted that the activation of STING pathway was more pronounced in G3 Terc^−/−^ mice than in Terc^+/−^ (G0) (Figure S3c‐d), implying age‐associated aggravation in the inflammatory response. Since influenza virus is a segmented RNA virus generally not to be recognized by the DNA‐sensing pathway, we speculated that STING pathway might be induced by intracellular DNA that was released upon dysfunctional telomeres. In support of this hypothesis, enhanced co‐localization of STING and DNA dye Draq5 (Benmerzoug et al., 2018) was observed in the lungs of Terc^−/−^ mice, particularly upon viral infection (Figure 1l). Together, our data confirmed that aberrant activation of the cGAS/STING pathway was induced in Terc^−/−^ mice during respiratory viral infection.

To further understand the functional relevance of the STING pathway, we next administrated a specific STING inhibitor, H151, in the established respiratory viral infection model. As expectedly, the administration of H151 largely abrogated the increased expression of SASP‐related cytokines and enhanced activation of STING pathway in Terc^−/−^ mice following IAV exposure (Figure S3e‐g). Viral loads were also decreased after H151 treatment (Figure S3h). Associated with this, the exacerbated inflammatory pathology in Terc^−/−^ mice, as characterized by reduced mortality, increased cellular infiltration, aggravated BALF protein leakage, and worsened histopathology, was substantially alleviated following H151 treatment (Figure 1m‐o). Taken together, our data indicated that Terc^−/−^ mice underwent the aging process with aberrant activation of the cGAS/STING pathway, resulting in enhanced inflammatory response and aggravated immunopathology following respiratory viral infection.

### 2.3 Terc^−/−^ macrophages adopt the senescence‐like phenotype and STING‐driven inflammatory property

**FIGURE 2:**
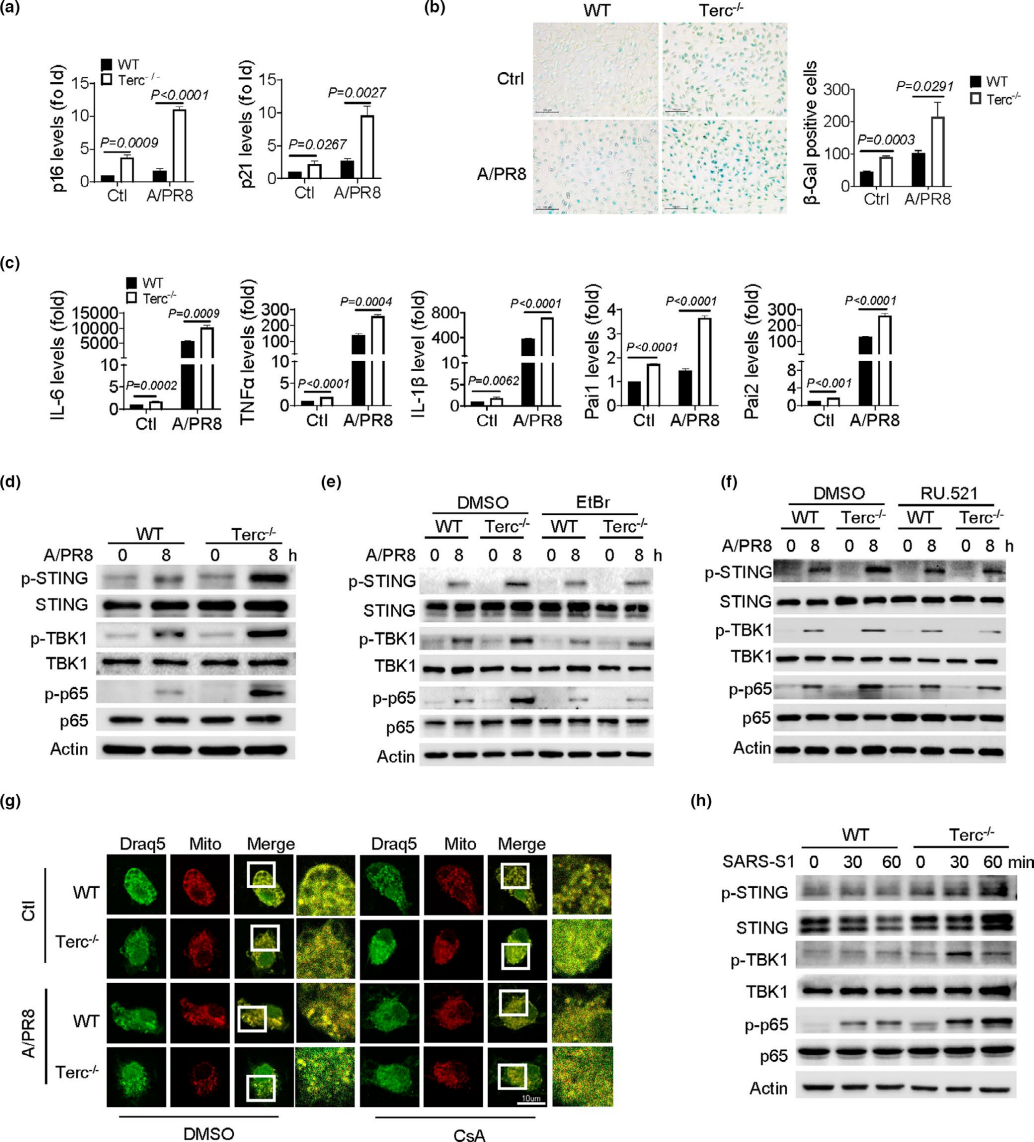
Terc^−/−^ macrophages display senescent‐like signatures and STING activation upon viral infection. (a‐d) BMDMs from WT and Terc^−/−^ mice were infected with IAV (A/PR8, MOI = 1) for 8 h. qPCR assay of p16 and p21 (a); staining of SA‐β‐Gal (b), Bars, 100 μm; qPCR assay of SASP genes as indicated (c); Immunoblotting of STING and the downstream signaling molecules as indicated (d). (e, f) Immunoblotting of STING and the downstream signaling molecules in BMDMs pretreated with EtBr (e) or RU.521 (f) or DMSO followed by A/PR8 infection. (g) Representative image showing leakage of mtDNA that were blocked by CsA treatment. The co‐localization of DNA dye Draq5 (cyan) and mitochondria (red) represents mtDNA in the mitochondria, and the green fluorescence scattered in the cytoplasm represents leaked mtDNA. Bars, 10 μm. (h) Immunoblotting of STING and the downstream signaling molecules in macrophages stimulated SARS‐CoV‐2 Spike (S1) (300 ng/ml) for the indicated time periods. Shown are representative images, and the data from three independent experiments are expressed as means ± SD

Macrophages are known as the first line of defense against pathogens, as well as the critical initiator and regulator of inflammatory responses. Indeed, our data showed that adoptive transfer of WT macrophages substantially improved the survival of Terc^−/−^ mice, highlighting the importance of macrophages with normal telomeres in maintaining tissue homeostasis (Figure S4a). To further understand the mechanism underlying telomere‐related immunopathology, we then conducted a comparative analysis of the phenotypic and functional traits of WT and Terc^−/−^ macrophages. Of interest, compared with WT control cells, bone marrow‐derived macrophages (BMDMs) from Terc^−/−^ mice expressed higher levels of canonical senescence markers p16 and p21. Moreover, β‐Gal staining was remarkably intensified in Terc^−/−^ BMDMs, particularly following IAV exposure (Figure 2a‐b). Along with this, the levels of SASP factors were elevated and viral stimulation further increased their production in Terc^−/−^ cells (Figure 2c). Thus, it appeared that macrophages with shortened telomeres underwent the senescence‐like transformation, particularly upon viral challenge.

Next, we examined the activation of the cGAS/STING pathway, the signaling proposed to be related with cellular senescence and inflammatory response. The result showed that phosphorylation of STING and the subsequent TBK1 and p65 was enhanced in Terc^−/−^ macrophages relative to WT cells following IAV challenge (Figure 2d). Notably, the augmented STING activation was largely abolished when cytosolic DNA was depleted by ethidium bromide (EtBr) (Figure 2e), the agent proposed to selectively deplete mtDNA without affecting genomic DNA. In parallel, the activated STING signaling pathway was significantly downregulated when cGAS was inhibited (Figure 2f). The data thus suggested that the cGAS/STING pathway was specifically activated in Terc^−/−^ macrophages upon sensing cytosolic DNA. Since leakage of mtDNA is frequently associated with cellular senescence, we then tested its potential involvement in this process by exploiting cyclosporin A (CsA), a well‐known destabilizer of mitochondrial permeability transition pore (mPTP) (Du et al., 2008). Not surprisingly, we observed a marked liberation of mtDNA into the cytosol in Terc^−/−^ macrophages following IAV stimulation, which was blocked by the disruption of mPTP via the application of CsA (Figure 2g). The data thus implied that STING activation in senescent macrophages was primarily mediated by mtDNA released into the cytosol.

Clinical data have demonstrated that hyperactivated monocyte‐macrophages, and excessive inflammatory response were causatively related with severe COVID‐19 in the aged (D'Agnillo et al., 2021; Nehme et al., 2020). Our above data implicated that STING pathway was excessively induced in senescent macrophages during respiratory viral infection. We thus wondered whether telomere‐related STING activation was implicated in SARS‐CoV‐2‐induced immunopathology. For the sake of biosafety, we herein utilized SARS‐CoV‐2‐spike protein (S1), a major component of the highly transmissible pathogen responsible for its binding, invasion, and hence infection. Indeed, administration of S1 protein caused remarkably enhanced activation of STING and the downstream NF‐KB and TBK1‐driven pathway in Terc^−/−^ macrophages relative to control cells (Figure 2h). Collectively, our data indicated that telomere‐defective macrophages tended to undergo the senescence‐like transformation, leading to cytosolic DNA liberation for STING activation and intensified inflammatory response following IAV or SARS‐CoV infection.

### 2.4 NLRP3 inflammasome is involved in STING‐mediated inflammatory response in senescent macrophages

**FIGURE 3:**
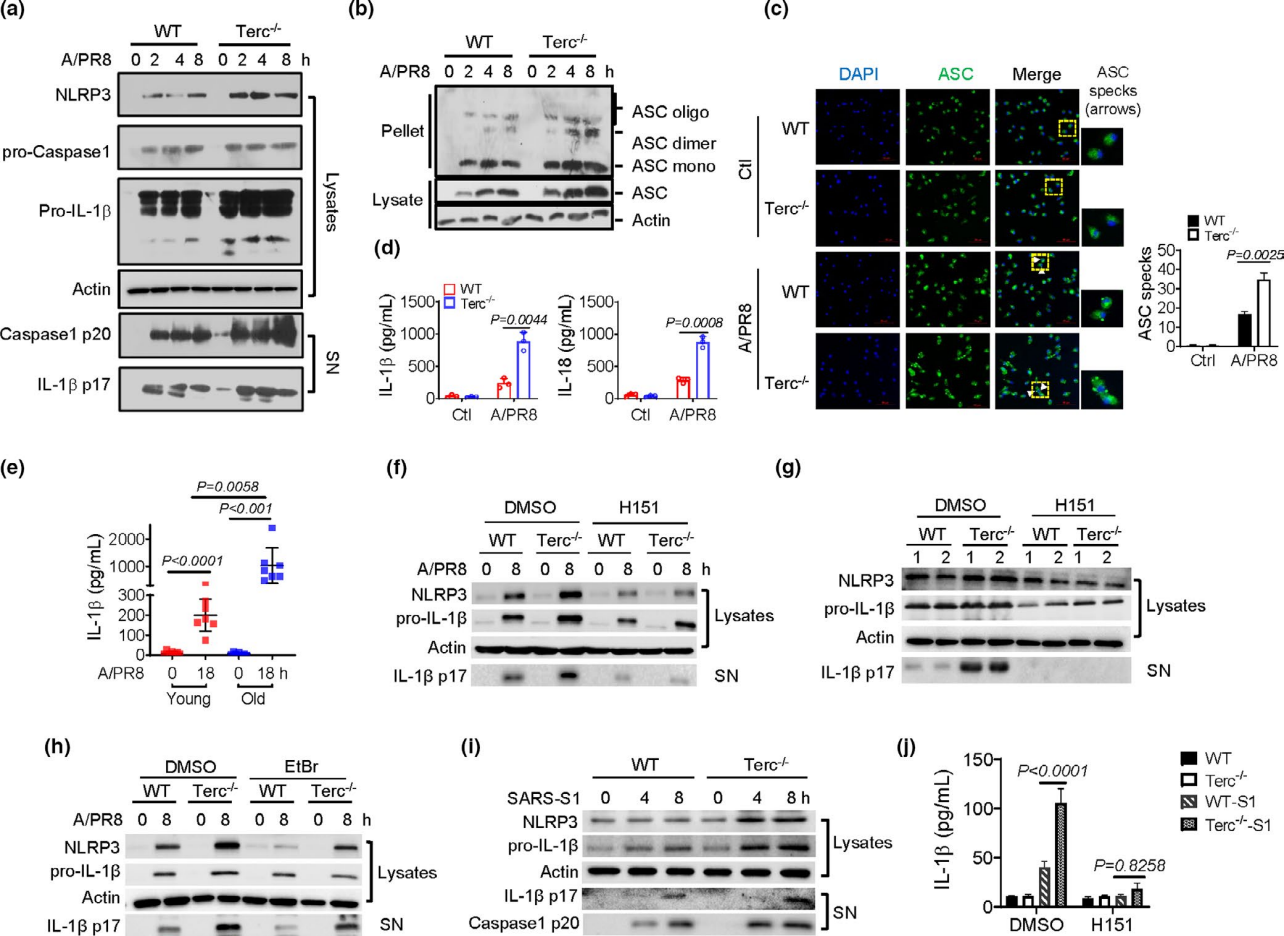
NLRP3 inflammasome is involved in STING‐driven inflammatory signaling in Terc^−/−^ macrophages. (a‐d) BMDMs from WT and Terc^−/−^ mice were infected with A/PR8 for 8 h. Immunoblotting of cleaved caspase‐1 and IL‐1β(a), SN, supernatants; Immunoblotting of ASC oligomerization in cross‐linked cytosolic pellets (b) and speck formation (c) of ASC, Bars, 50 μm; ELISA analysis of IL‐1β and IL‐18 secretion (d). (e) ELISA analysis of IL‐1β released by PBMCs from young (<30 y) or old (>60 y) healthy subjects upon A/PR8 stimulation for 18 h. (f) Immunoblotting of cleaved IL‐1β in macrophages pretreated with H151 (1 μM) or DMSO followed by A/PR8 stimulation. (g) Immunoblotting of cleaved IL‐1β in lungs from WT and Terc^−/−^ mice that were pretreated with H151 or DMSO and subjected to A/PR8 infection for 48 h. (h) Immunoblotting of cleaved IL‐1β in macrophages pretreated with EtBr or DMSO followed by A/PR8 infection. (i) Immunoblotting of caspase‐1 and IL‐1β cleavage in macrophages stimulated with SARS‐CoV‐2 Spike protein (S1) for the indicated time periods. (j) ELISA analysis of IL‐1β released by macrophages pretreated with H151 or DMSO followed by stimulation of SARS‐CoV‐2 S1 protein. Shown are representative images and the data from three independent experiments are expressed as means ± SD

NOD‐, LRR‐, and pyrin domain‐containing protein 3 (NLRP3) is a general sensor of cellular damage and stress to initiate the inflammasome pathway and inflammatory response. The activation of NLRP3 inflammasome has been implicated in the aging and aging‐associated diseases including IAV and COVID‐19 pathogenesis (Xian et al., 2021). We therefore proceeded to assess the potential role of NLRP3 inflammasome in telomere‐related immunopathology during respiratory infection. Remarkably, the activation of the NLRP3 inflammasome, as demonstrated by the induction of NLRP3 and the cleavage of caspase‐1 and IL‐1β, was enhanced in Terc^−/−^ macrophages relative to WT cells following IAV infection (Figure 3a). Moreover, the oligomerization of and the speck formation of apoptosis‐associated speck‐like protein containing a CARD (ASC), indicative of NLRP3 activation, were boosted in those cells (Figure 3b‐c). Consequently, Terc^−/−^ macrophages demonstrated to produce higher level of IL‐1β and IL‐18 compared with WT cells (Figure 3d). The data thus revealed the enhanced activation of NLRP3 inflammasome in senescent macrophages upon viral infection.

Since dysfunctional telomeres have been recognized as a general signature of cellular senescence, we thus wondered whether the observed inflammasome enhancement would be reproduced in aged human cells. To this end, peripheral blood monocytes (PBMCs) from healthy subjects of different ages were collected and subjected to viral infection. The data showed that PBMCs from aged people (>60 years) produced a higher level of IL‐1β compared with that from young individuals (<30 years) on stimulation of IAV or VSV (Figure 3e and Figure S5a). Along with this, the expression of proinflammatory cytokines IL‐6 and TNFα was elevated in aged PBMCs upon infection (Figure S5b). More importantly, we found that telomere lengths in PBMCs were inversely correlated with the levels of IL‐1β, IL‐6, and p16 (Figure S5c) implying that shortened telomeres might be indicative of the inflammaging. In agreement, the activation of STING pathway was significantly enhanced in aged PBMCs post IAV infection (Figure S5d). Combined, the data indicated that the activation of NLRP3 inflammasome was reinforced in aged innate immune cells following respiratory viral infection.

Since the cGAS/STING pathway has a vital role in sensing intracellular cellular stress and subsequently activating the inflammatory signaling, we wondered whether the enhanced NLRP3 inflammasome observed in Terc^‐/‐^ macrophages was integral into the STING‐driven pathway. Indeed, the blockade of STING pathway with H151 or the depletion of cytosolic DNA with EtBr led to a remarkable decrease the level of cleaved IL‐1β in Terc^−/−^ macrophages or mice upon IAV infection (Figure 3f‐h). Likewise, SARS‐CoV‐2 S1 induced boosted activation of NLRP3 inflammasome and IL‐1β release in Terc^−/−^ macrophages relative to WT cells, which, however, was markedly repressed on STING inhibition (Figure 3i). Accompanied with this, the production of IL‐6 and TNFα was also repressed (Figure 3j and Figure S5e). We thus proposed that NLRP3 inflammasome, downstream of STING, was highly activated and contributed to the exaggerated inflammation in Terc^−/−^ macrophages during IAV or SARS‐CoV‐2 infection.

### 2.5 Viral infection aggravates mitochondria distress and mtDNA leakage underpinning STING activation in Terc^−/−^ macrophages

**FIGURE 4:**
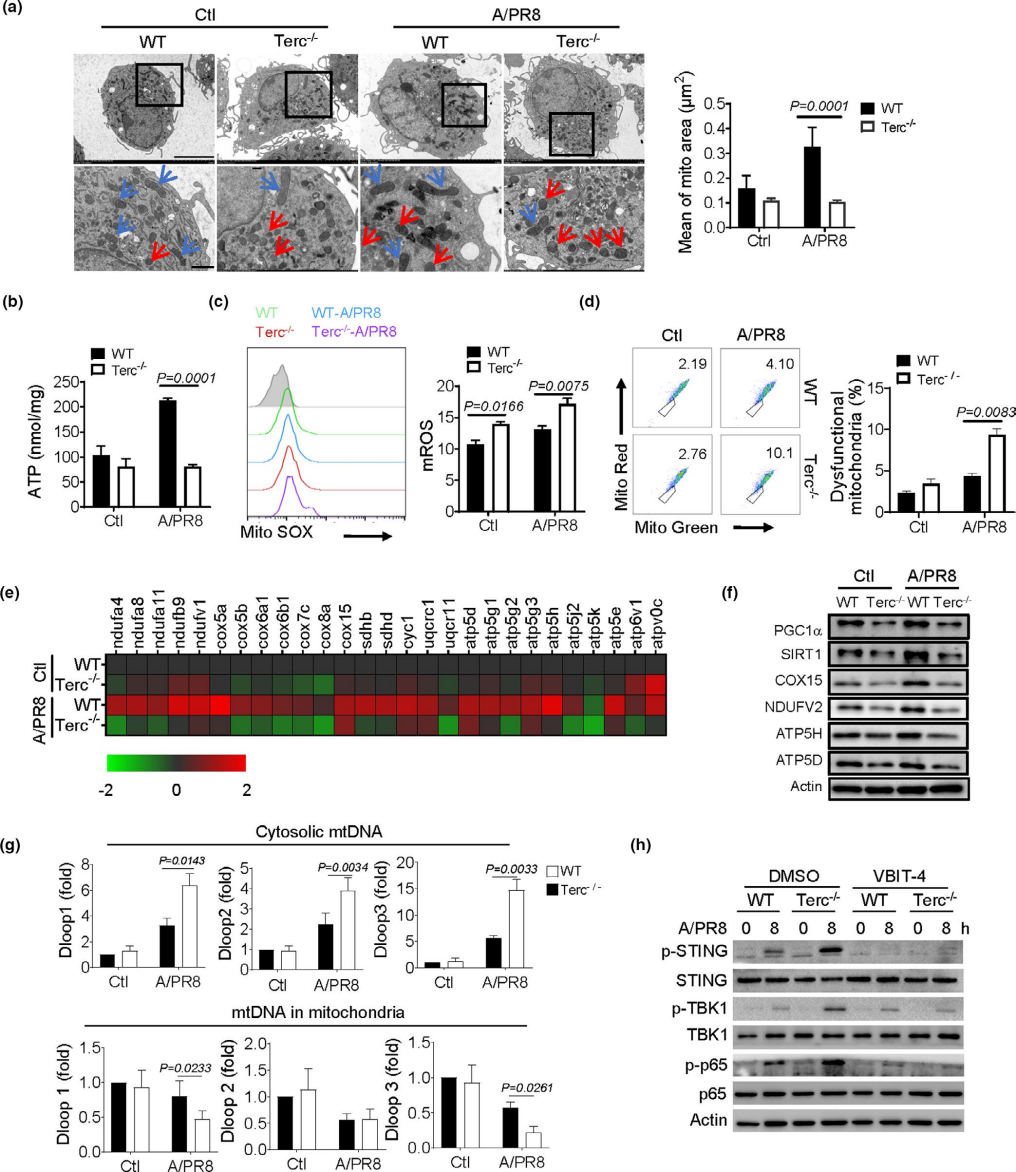
Viral infection aggravates mitochondria distress and promotes mtDNA liberation for STING activation in senescent macrophages. (a‐f) BMDMs from WT and Terc^−/−^ mice were infected with A/PR8 for 8 h and then subjected to functional analysis. Representative transmission electron micrographs (TEM) showing mitochondrial amounts and morphology (a). The red arrow marks unhealthy mitochondria and blue arrow marks healthy mitochondria; Assay of ATP generation (b); Flow cytometry and quantification of mitochondrial ROS levels (c); Flow cytometry of dysfunctional mitochondria (d); Heatmap showing the expression of representative mitochondrial respiratory genes by qPCR (e); Immunoblotting of mitochondria‐associated molecules (f); (g) qPCR quantification of representative mtDNA genes in cytosol and mitochondria respectively. (h) Immunoblotting of STING and the downstream signaling molecules in macrophages pretreated with VBIT‐4 or DMSO followed by A/PR8 infection. The results are from three independent experiments. Shown are representative images, and the data are expressed as means ± SD

Mitochondria is known as a central cellular organelle and a key signaling hub regulating a network of cellular activities such as cellular metabolism, proliferation, apoptosis, senescence, and immune responses. However, age‐dependent decline in mitochondrial integrity profoundly affects cellular physiology and signaling pathway, and hence induces pathologic inflammation. We thus proceeded to examine the mitochondrial status in Terc^−/−^ macrophages and evaluate its relevance to STING‐mediated immunopathology. We initially observed that the amount and morphology of mitochondria were altered in Terc^−/−^ macrophages following IAV exposure. As shown in Figure 4a, WT cells possessed healthy mitochondria with rod‐shape and organized cristae, whereas Terc^−/−^ macrophages harbored mitochondria characterized by swollen shape and irregular rarefied cristae. Consistently, Terc^−/−^ macrophages displayed compromised ATP generation but increased mROS stress compared with control cells (Figure 4b‐c). A greater quantity of dysfunctional mitochondria, as defined by Mito‐Green^pos^/Red^neg^ staining, was observed in Terc^−/−^ macrophages particularly following IAV exposure (Figure 4d). The expression of the genes representative of mitochondrial respiratory chain complex (Cox) I‐IV was consistently downregulated in Terc^−/−^ macrophages relative to WT control cells (Figure 4e). Moreover, the protein level of the key factors essential for mitochondrial biogenesis and homeostasis, such as peroxisome proliferator‐activated receptor‐γ coactivator (PGC)‐1α and sirtuin 1 (SIRT1), was decreased in Terc^−/−^ macrophages (Figure 4f). The data thus revealed that mitochondria in Terc^−/−^ macrophages became defective in mitochondrial integrity, function, and biogenesis.

Since mitochondria distress, particularly the leaked mtDNA might serve as the endogenous signal for STING activation, we then examined the potential role for dysfunctional mitochondria in telomere‐related immunopathology. By quantifying mtDNA in different cellular compartments (Yu et al., 2020), we revealed that the amount of mtDNA released into the cytosol was boosted in Terc^−/−^ macrophages following IAV infection. Conversely, the amount of mtDNA remaining in mitochondria was reduced in Terc^−/−^ macrophages (Figure 4g), further substantiating the translocation of mtDNA from mitochondria to the cytosol. In addition, our above results demonstrated that Terc^−/−^ macrophages were characterized by increased oxidative stress and abundant mROS generation (Figure 4c), which were thought to expedite mtDNA release through oligomerized voltage‐dependent anion channel 1 (VDAC1) (Kim et al., 2019). We therefore tested the effect of VDAC1 inhibition on mtDNA liberation using the specific inhibitor, VBIT‐4. Expectedly, VBIT‐4 treatment remarkably hindered STING activation in Terc^−/−^ macrophages relative to WT cells upon IAV infection (Figure 4h). Taken together, our data indicated that Terc^−/−^ macrophages yielded dysfunctional mitochondria particularly upon viral infection, leading to mtDNA leakage for STING activating and the inflammatory response.

### 2.6 Treatment of senolytics alleviates the burden of senescent cells, mitochondrial abnormity, and STING activation during respiratory viral infection

**FIGURE 5:**
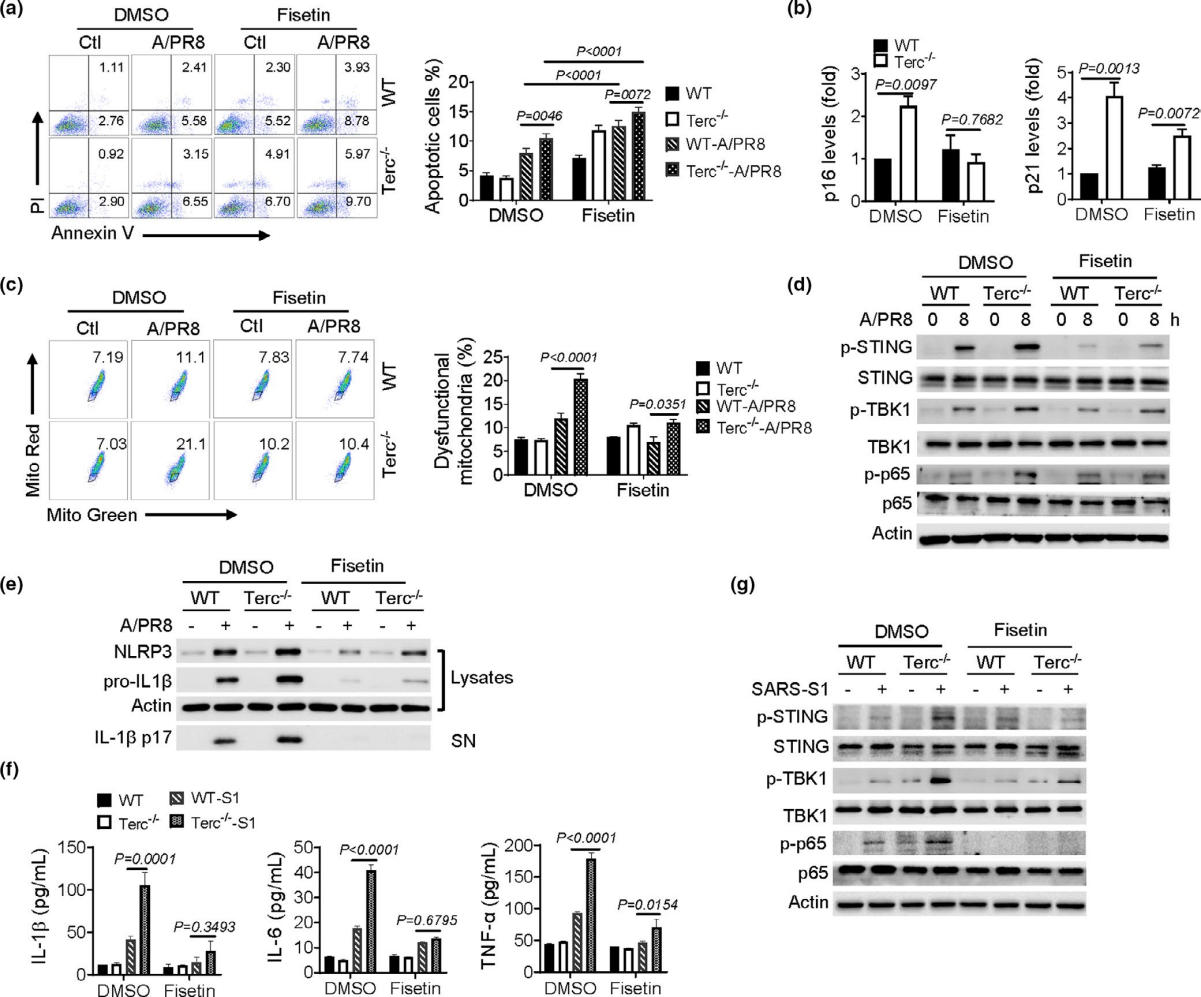
Senolytics reduces mitochondrial stress and STING activation in Terc^−/−^ macrophages following viral infection. (a‐f) BMDMs from WT and Terc^−/−^ mice were pretreated with fisetin (30 μM) or DMSO and then subjected to A/PR8 infection or not. Annexin V/PI staining and flow cytometry of apoptotic rate of macrophages (a); qPCR analysis of the expression of p16 and p21 normalized to DMSO WT Ctrl (b); Flow cytometry of dysfunctional mitochondria by staining with Mito‐Green/Mito‐Red (c); Immunoblotting of STING and the downstream signaling molecules (d). Immunoblotting of NLRP3 and cleaved IL‐1β (e). (f) ELISA analysis of the indicated cytokines, and (g) Immunoblotting of STING and the downstream signaling molecules in macrophages pretreated with fisetin or DMSO followed by stimulation of SARS‐CoV‐2 S1. Shown are representative images and the data from three independent experiments are expressed as means ± SD

Recent studies report that elimination of senescent cells, namely senolysis, can potentially lessen age‐associated pathology and extend lifespan in aged mice (van Deursen, 2019). As dysfunctional telomeres were causatively related with cellular senescence and organismal aging, we wondered whether telomere‐associated immunopathology would be improved by reducing the burden of senescent cells using senolytic agents. For this, a well‐appreciated senolytics, fisetin (Camell et al., 2021; Yousefzadeh et al., 2018), was applied to WT and Terc^−/−^ macrophages prior to IAV infection. Remarkably, the administration of fisetin increased cellular apoptotic rate in Terc^−/−^ macrophages. The level of senescent markers p21 and p16 was substantially decreased presumably due to the elimination of senescent cells by senolytic agent (Figure 5a‐b). Moreover, fisetin treatment reduced the percentage of dysfunctional mitochondria (Mito‐Red^neg^/Mito‐Green^pos^) in Terc^−/−^ macrophages following IAV exposure (Figure 5c). Therefore, enhanced activation of STING and subsequent NLRP3 inflammasome were lessened upon fisetin treatment (Figure 5d‐e). Likewise, SARS‐S1‐induced STING activation, as well as the secretion of IL‐1β, IL‐6, and TNFα were suppressed upon fisetin administration (Figure 5f‐g). Thus, our data indicated that senolytic treatment, likely through inducing cellular apoptosis and reducing loads of dysfunctional mitochondria, substantially suppressed the activation of STING signaling and the inflammatory response in senescent macrophages during IAV or SARS‐CoV‐2 infection.

### 2.7 Senotherapeutics improves viral pneumonia in Terc^−/−^ mice

**FIGURE 6:**
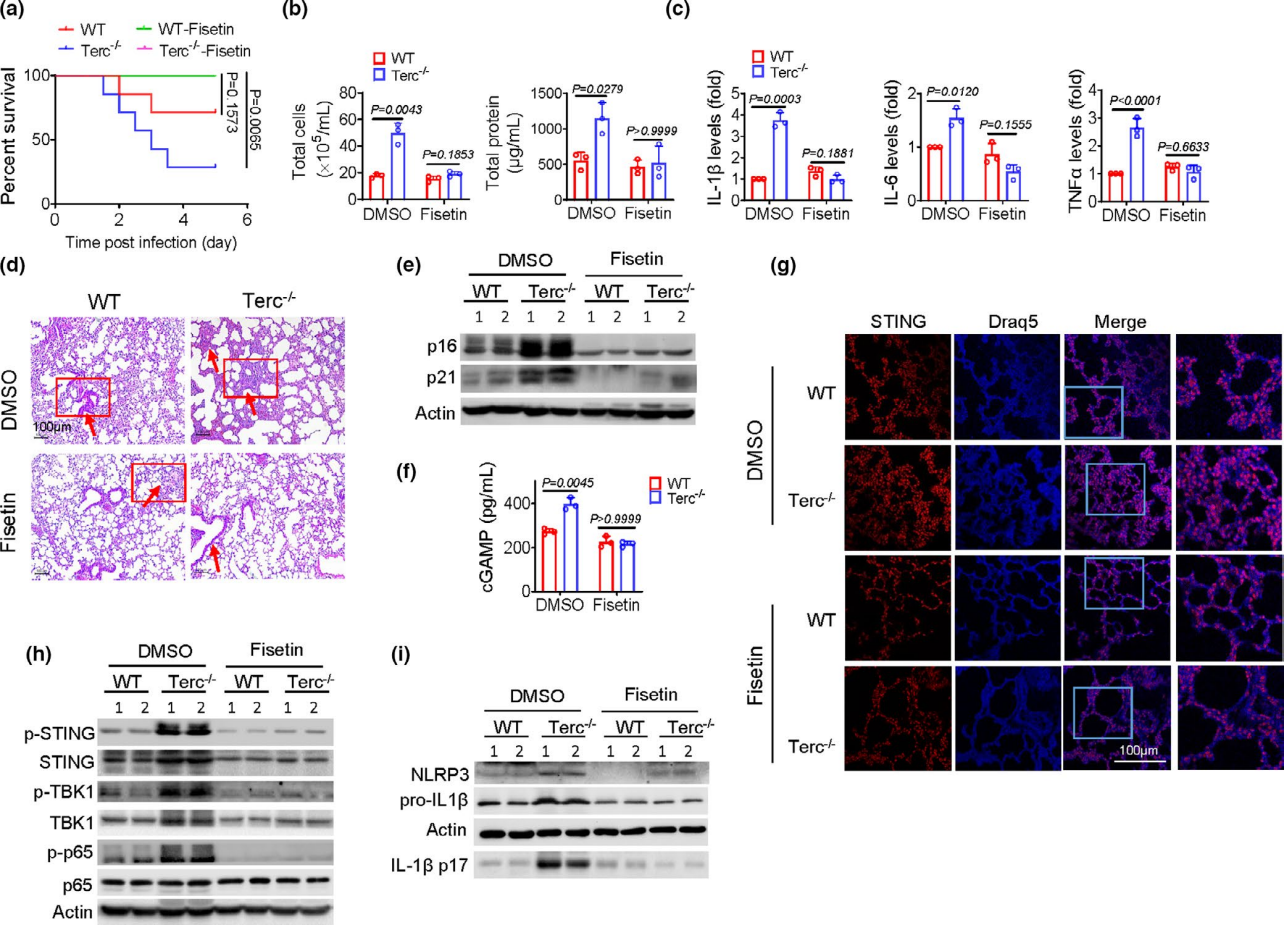
Senolytics confers the protection for Terc^−/−^ mice against viral pneumonia. (a) WT and Terc ^−/−^ mice (*n *= 7) were pretreated with fisetin (50 mg/kg) or DMSO for 3 days, followed by A/PR8 infection. Animals were monitored every 10 h for survival rate. WT and Terc ^−/−^ mice (*n *= 3) were pretreated with fisetin or DMSO, followed by A/PR8 infection (1 × 10^4^ pfu/mouse) for 48 h. (b) Counts of total cells and protein leakage in BALF; (c) Relative levels of proinflammatory cytokines in Lungs; (d) Representative H&E staining of lung tissues; (e) Immunoblotting of p16 and p21 in lung tissues; (f) BALF level of cGAMP; (g) Confocal staining of DNA dye Draq5 (cyan) and STING (red) in lungs; (h, i) Immunoblotting of lung lysates for STING and the downstream signaling molecules (h), and for NLRP3 and cleaved IL‐1β (i). The results are from one of two independent experiments. Shown are representative images, and the data are expressed as means ± SD

Inspired by the above findings, we proceeded to test the in vivo effect of senolytics in protecting Terc^−/−^ mice against respiratory infection. To this end, a single dose of fisetin (50 mg/kg) was administrated into mice prior to IAV infection (Camell et al., 2021). As shown in Figure 6a‐d, fisetin treatment markedly improved the survival in Terc^−/−^ mice following IAV exposure. Associated with this, the exaggerated lung inflammation and injury were relieved, as evidenced by a profound reduction in inflammatory cells infiltration, BALF protein leakage, proinflammatory cytokines production, and lung histopathology. Concurrently, lung expression of senescence signature genes p16 and p21 in Terc^−/−^ mice was decreased upon fisetin treatment (Figure 6e). Viral loads, however, were not markedly altered upon fisetin treatment (Figure S6a). Likewise, fisetin had no significant effect on viral burdens in murine BMDMs or human epithelial cells (A549) (Figure S6b), indicating that the protective effect of fisetin was not due to its direct antiviral property. Notably, fisetin treatment caused a reduction in BALF level of cGAMP in Terc^−/−^ mice post viral infection, implying that the primed signal for STING activation was reduced (Figure 6f). In support of this, co‐localization of mtDNA and STING in lung tissues was reduced upon fisetin administration (Figure 6g), and the activation of STING pathway and NLRP3 inflammasome was accordingly lessened (Figure 6h‐i).

To further corroborate the effect of senolytics on telomere‐related immunopathology, we additionally utilized another well‐established senolytic drug combination, dasatinib (D) plus quercetin (Q). As shown in Figure S7a‐c, D+Q treatment markedly reduced SASP molecules expression, lung inflammatory pathology, and the STING/NLRP3 pathway activation in Terc^−/−^ mice following IAV infection. Together, we showed that the senolytic agents conferred a profound protection for aged mice against respiratory viral infection primarily through reducing burdens of senescent cells, improving mitochondria integrity, and hence dampening STING‐mediated pathogenic inflammation.

## 3 DISCUSSION

Despite accumulating evidences supporting the critical involvement of immune deregulation in age‐associated exacerbation in respiratory viral infection, it is still unknown whether the aggravated inflammation is more dependent on a specific immune pathway and whether interference of such a dominant mechanism would bring health benefits. Using the late generation of Terc^−/−^ mice, we confirm that the aged animals are exquisitely vulnerable to IAV and SARS‐Cov‐2 infection, primarily through aberrant activation of the cGAS/STING pathway induced by mitochondrial disintegration. In particular, we show that Terc^−/−^ macrophages adopt senescence‐like phenotype and induce mitochondrial distress facilitating mtDNA leakage, which in turn triggers the cGAS/STING pathways and NLRP3 inflammasome activation, and thereby predisposes Terc^−/−^ mice severe disease during normally non‐lethal infection. Since the late generation of Terc^−/−^ mice have long been established as a prototypic aging model, the present study links the immunosenescence with exacerbated respiratory infection and provides a plausible explanation for the increased susceptibility of the aged to severe respiratory infection such as SARS‐CoV‐2 or IAV. More importantly, our data demonstrate that the treatment of senolytics substantially reduced the burden of senescent cells, improved mitochondrial integrity, alleviated STING‐mediated inflammatory pathway, and thereby conferred a profound protection for aged (Terc^−/−^ G3) mice against respiratory infection. The findings are of clinical relevance since currently no specific therapeutics are available for critical respiratory infections like COVID‐19, particularly for the older population.

As aging is a gradually developing process impinged by a broad spectrum of factors involving genetic, environmental, ethnic, and nutrimental elements, it seems inappropriate to apply biological ages to define the senescent status of immune cells. Instead, telomere length, an excellent measure of cellular proliferative history and replicative reserve, might be a more precise indicator of cellular functional senescence (Blasco et al., 1997; Chakravarti et al., 2021; Sahin et al., 2011; Sanchez‐Vazquez et al., 2021). In support of this notion, a recent study showed that people born with abnormally short telomeres have short‐lived immune cells, sharing characteristics of cells in older people without telomere disorder (Wagner et al., 2018). Moreover, clinical data revealed a correlation between shortened telomeres and hosts’ vulnerability to viral infection (Cohen et al., 2013). Age‐dependent telomere shortening in hematopoietic cells (HCTL) was reported to coincide with a steep increase in COVID‐19 mortality (Sanchez‐Vazquez et al., 2021), indicating that telomere conditions have predictive value in assessing the individual's susceptibility to respiratory infection regardless of his/her age. For these reasons, our current study exploited Terc^−/−^ mice rather than biologically aged mice to examine the impact of aging on the hosts’ response to respiratory viral infection. We attached a particular attention to monocyte‐macrophages because this immune cell population demonstrated to have a central role in the development and progression of respiratory viral infection such as COVID‐19 (Iwasaki & Pillai, 2014; Schultze & Aschenbrenner, 2021).

Of note, our study demonstrates that macrophages from Terc^−/−^ mice acquired senescent‐like phenotype, characterized by shortened telomeres, increased expression of senescent markers p16 and p21, and secretion of senescence‐associated inflammatory cytokines. Furthermore, the senescent‐like macrophages revealed to accumulate dysfunctional mitochondria with impaired respiratory activity and increased permeability, which in turn caused cytosolic mtDNA deposit and activated the cGAS/STING pathway and NLRP3 inflammasome. It appears that macrophages with dysfunctional telomere are primed to be more easily and highly incited upon infection. We thus propose that age‐dependent decline in mitochondrial quality control may have a causative role in aggravated respiratory infection in the elderly. In support of this, the level of mtDNA in the tissues and the circulation were recently reported to increase with age and correlate with the severity of diseases such as COVID‐19 (Smith et al., 2020; Tumburu et al., 2021). Although the mechanistic link between mitochondrial instability and telomere deficits remains elusive currently, studies from us and other researchers revealed that the master factors PGC‐1α and TNFAIP3 might act downstream of the telomere and p53 to regulate the genes essential for mitochondrial integrity (Sahin et al., 2011). Other studies demonstrated that telomerase reverse transcriptase (TERT) directly localized in mitochondria and modulated mitochondrial activity (Ahmed et al., 2008). Future studies might be merited to further address how mitochondria and telomeres interact and control the immune homeostasis during aging.

The currently ongoing respiratory infections are mostly from RNA viruses such as IAV and SARS‐CoV‐2, which are thought to be sensed by the RNA‐sensing (MDA/RIG‐I) pathway rather than the DNA‐sensing (cGAS/STING) system. However, emerging data have shown that the infectious agents might, through directly or indirectly acting on mitochondria, participate in the STING‐mediated pathway (Hanada et al., 2020). The strength and duration of STING pathway appear to be determined by both cellular intrinsic signals and pathogen‐derived cues converging at the mitochondria. Although the impact of aging on mitochondrial biology is multifaceted, our data reveal that the leaked mtDNA and the resultant aberrant STING activation play a central role in telomere‐related immunopathology, as blockade of mtDNA leakage or depletion of cytosolic mtDNA lessened the inflammatory response in Terc^−/−^ macrophages following influenza infection. Moreover, specific inhibition of the STING pathway conferred a profound protection against severe viral pneumonia for Terc^−/−^ mice, bolstering the clinical observation that the STING‐mediated DNA‐sensing pathway is critically involved in the pathogenesis of inflammatory lung diseases such as COVID‐19 (Benmerzoug et al., 2018). Interestingly, a recent study showed that bats harbored a non‐functional STING gene, which allowed them to withstand tissue stress and SARS‐CoV‐2 infection without ill effects (Gorbunova et al., 2020). Inspired by these findings, scientists endeavor to develop the therapeutics aimed to improve mitochondrial integrity or block the STING pathway in treating inflammatory diseases (Hong et al., 2021).

Our present study show that the pathogenic role of STING in respiratory infection also involves aberrant activation of NLRP3 inflammasome, an essential pathway critically implicated in inflammaging and age‐associated disorders. Indeed, NLRP3 inflammasome pathway has been identified as a risk factor for severe pulmonary infection caused by IAV and COVID‐19 (Lara et al., 2020). Although multiple signals such as mROS, ATP, or calcium are able to trigger the inflammasome pathway, our study indicates that mtDNA‐dependent STING activation precedes the NLRP3 pathway in Terc^−/−^ macrophages, as the inhibition of STING or elimination of mtDNA profoundly reduced NLRP3 induction and IL‐1β production. In support of this, Wang et al. reported that STING physically interacted with NLRP3 and promoted NLRP3 activation via removing the polyubiquitination (Wang et al., 2020). Alternatively, the protein STING acted through the lysosome to induce potassium efflux and hence accelerated NLRP3 activation (Gaidt et al., 2017). Based on these findings, strategies to ameliorate the inflammatory pathology during respiratory infection such as COVID‐19 are now designed to target STING rather than the downstream NLRP3 for more health benefits.

Of interest, the senescent immune system was recently reported to have a crucial role in promoting systemic aging (Yousefzadeh et al., 2021), and pharmacological or genetic clearance of senescent immune cells was shown to clinically prolong lifespan and ameliorate age‐related pathologies. To treat inflammatory lung diseases associated with aging, much energy is invested in searching specific agents to inhibit or neutralize the inflammatory effector factors. Since aging is generally an irreversible and progressive process with an increasing deposit of stressed products such as mtDNA, blocking the inflammatory signaling, or effector molecules only has temporary and limited advantage, when compared with senotherapeutics that are aimed to eliminate the source of inflammation by directly targeting senescent cells (Camell et al., 2021). Supportively, our data indicated that administration of a well‐established senotherapeutic agent, fisetin, remarkably reduced inflammatory responses in Terc^−/−^ macrophages upon IAV or SARS‐Cov‐2 infection. The effect is likely due to alleviation of the burden of senescent‐like macrophages that are characterized by shortened telomeres, damaged mitochondria, mtDNA leakage, and hence aberrant STING/NLRP3 activation. Interestingly, during our preparation of this manuscript, Robbins and Kirkland et al. reported that senotherapeutics significantly reduced mortality and inflammatory markers of aged individuals following coronavirus infection, further supporting the potential of the senolytics in treating age‐associated respiratory infection (Camell et al., 2021). The discovery is of critical importance because respiratory tract infections are among the leading causes of death in the elderly, and no effective therapeutics are available yet.

Despite current knowledge about cellular senescence is mainly derived from somatic cells, the present study is focused on macrophages because this population of innate immune cells are increasingly prove to have a central role in inflammaging and age‐associated pathology. Macrophage‐targeted senotherapeutics is currently underway to treat age‐related disorders including critical respiratory infection (Pham et al., 2021). Another rationale for the macrophage‐centered study is the so called “trained immunity”, a non‐specific but stable immunological memory formed by innate immune cells after initial infection (You et al., 2021). Emerging evidences have shown that early infections, through metabolic‐epigenetic rewiring, shape long‐lasting immunological imprinter in innate immune cells, which endows the immune cells such as macrophages to rapidly and highly respond to re‐infection, and may simultaneously induce the aggravated inflammatory response. For this reason, targeting “memory macrophages” that are expected to be proficient in the aged population may have extra benefits to avoid the accumulative adverse effect with aging. In this regard, further study might be needed to identify the cells “old enough” to be eliminated, and the appropriate parameters to define the immunosenescence such as telomere length, mitochondrial status, and intrinsic STING activity merit further investigation.

In conclusion, we show that mice with dysfunctional telomeres are increasingly susceptible to respiratory viral infection primarily through mitostress‐driven cGAS/STING activation. The senotherapeutics may be a promising approach to treat severe respiratory infection, particularly for the elderly population.

## 4 METHODS AND MATERIALS

### 4.1 Ethics statement

This study was conducted according to the principles expressed in the Declaration of Helsinki. Ethics approval was obtained from the Research Ethics Committee of Affiliated Hospital of NUCM (2018NL165‐02).

### 4.2 BMDM generation

Bone marrow (BM) cells isolated from 8‐week‐old WT or Terc^−/−^ male mice were cultured in complete DMEM medium supplemented with 25 ng/ml M‐CSF (PeproTech). On the seventh day, BMDMs were harvested for the subsequent analysis.

### 4.3 Influenza virus infection

In vivo, WT or Terc^−/−^ male mice (8–12 weeks‐old) were intranasally inoculated with IAVs (A/PR8) in a total volume of 50 μl (25 μl in each nasal cavity) under light anesthesia (3% isoflurane). Control (uninfected) mice were treated intranasally with PBS. Non‐lethal dose of virus (1 × 10^4^ PFU/mouse) was used for functional study. For the mortality studies, the mice were intratracheally instilled with 1 × 10^8^ PFU/mouse of A/PR8. For the adoptive transfer experiment, BMDMs (2 × 10^6^ / mouse) were administered intravenously into Terc^−/−^ mice at −2, 1, and 3 d during IAV infection.

### 4.4 Immunofluorescence microscopy

Frozen sections of lung tissue were placed at room temperature for 30 min, lung cells were fixed with 4% PFA for 15 min, permeabilized with 0.2% Triton X‐100 for 10 min, then blocked with 3% BSA for 1 h and washed 3 times in PBS. The lung tissues were then incubated with rabbit anti‐STING (1/300) overnight at 4°C, washed and incubated with Goat anti‐Rabbit IgG (H+L) Alexa Fluor 568 secondary antibody for 1 h at room temperature. After washing 3 times, lungs were incubated with DNA dye Draq5 (1/1000) for 5 min, washed and quenched before mounting. Finally, tissues were observed using Leica TCS SP8 laser scanning confocal microscope (Leica Microsystems Ltd., Wetzlar, Germany). Images were analyzed using LAS X software.

In vitro, BMDMs were harvested and placed onto cell slides overnight at 37°C 5% CO2 in DMEM complemented medium. Cells were fixed with 4% PFA for 20 min, permeabilized with 0.2% Triton X‐100 for 5 min, blocked with 3% BSA for 1 h, washed with PBS, and incubated with MitoTracker Red (500 nM) for 45 min at 37 °C. After washing, cells were stained with DNA dye Draq5 (1/1000) for 5 min to label dsDNA and the anti‐fluorescence quencher was added before mounting. Cells were then visualized via Leica TCS SP8 laser scanning confocal microscope.

### 4.5 SA‐β‐gal staining

In situ β‐galactosidase Staining Kit (Beyotime) was used to detect cell senescence. In vitro, BMDMs were placed onto cell slides and infected with A/PR8 for 8 h. Cells were fixed with 4% PFA for 10 min. After washing with PBS, cells were stained with SA‐β‐gal detection solution at 37 °C overnight, washed with PBS, and observed under the microscope. In vivo, frozen sections of lung tissue were fixed with 4% PFA for 15 min, washed with PBS for 3 times, stained and analyzed as above.

### 4.6 Mitochondrial DNA depletion

On the seventh day, BMDMs were collected and cultured for 4 days in the presence of EtBr (200 ng/ml), washed, and stimulated with IAV for 8 h.

### 4.7 Mitochondrial and cytoplasmic mtDNA detection

In order to detect the leakage of mtDNA, mitochondria and cytoplasm were isolated using the cell mitochondrial isolation kit (Beyotime). In short, BMDMs were incubated in 1 ml mitochondrial isolation buffer on ice for 10 min followed by homogenizing with a glass homogenizer on ice for 20 passes. The cell lysate was centrifuged at 600g, 4°C for 10 min, and the supernatant was centrifuged at 11,000g for 10 min at 4°C to obtain mitochondria (precipitate) and cytoplasm (supernatant). Total DNAs of mitochondria and cytoplasm were extracted using the FlexiGene (QIAGEN) according to the manufacturer's instructions. The primers against mtDNA and genomic DNA were listed in Table S1.

### 4.8 Statistical analysis

All of the data, unless otherwise indicated, are presented as the means ± SD of three independent experiments. The statistical significance of the differences between two groups was analyzed with Student's t‐test. Multiple group comparisons were performed by two‐way ANOVA followed by Bonferroni post hoc t‐test. The Kaplan–Meier survival analysis with a log‐rank test was applied to evaluate the survival curve. All of the calculations were performed using the Prism software program for Windows (GraphPad Software). A *p*‐value of 0.5 or less was considered statistically significant.

## CONFLICT OF INTERESTS

The authors declare no competing interests.

## AUTHOR CONTRIBUTIONS

L.S. conceived the experiments, and supervised the study. N.L., Y. Z., and X.L. performed the experiments, analyzed the data, and prepared the manuscript. L. Y., Z. L., Y. K., Y. D., and N. K. helped conduct experiments and managed mouse breeding. W. W. provided blood samples. L.S., N. K., and N. L wrote the manuscript.

## Supporting information

Supplementary Material

## Data Availability

The data are available from the corresponding author upon reasonable request.
