# Supplementary material for: Dysfunctional telomeres through mitostress‐induced cGAS/STING activation to aggravate immune senescence and viral pneumonia
Source: Aging Cell. 2022 Mar 21;21(4):e13594. doi: 10.1111/acel.13594 (PMC9009109; doi:10.1111/acel.13594)
Supplement: Supplementary file 1 — Supplementary Material [file ACEL-21-e13594-s001.docx]

**Supplementary Methods and Figures**

**
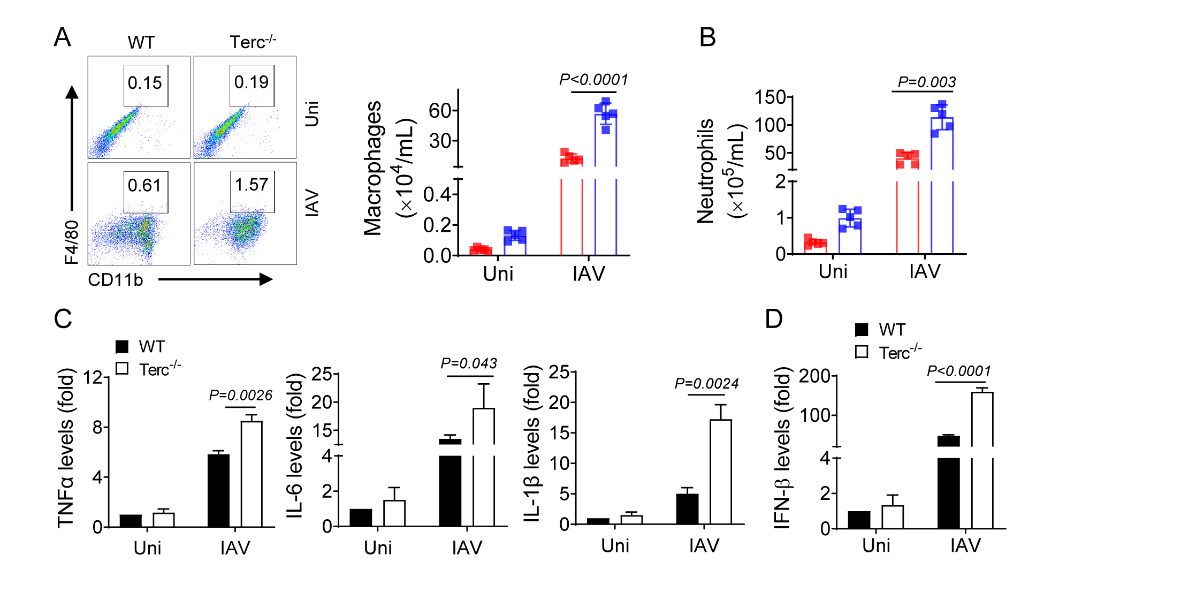
**

**Figure S1. Cellular infiltration and cytokines production in IAV-infected murine lungs.** WT and Terc ^-/-^ mice were administrated as described in Figure 1B. **(A-D)** Flow cytometry analysis of macrophages (A) and neutrophils (B) in BALF; qPCR quantification of proinflammatory (C) and antiviral cytokines (D). The data from one of three independent experiments are expressed as means ± SD.


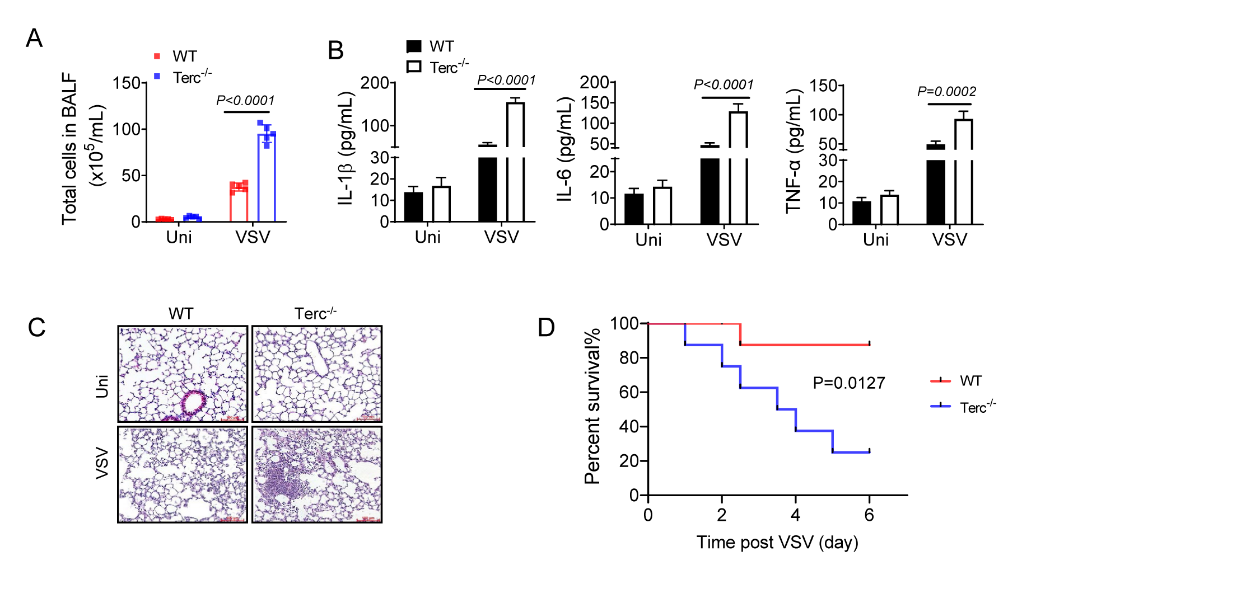


**Figure S2. Dysfunctional telomeres aggravate VSV-induced lung inflammation and injury.** WT and Terc ^-/-^ mice (n=5) were infected with VSV (1x10^4^ pfu/mouse) for 48 h, and then sacrificed for functional analysis. **(A)** Counts of total cells recovered from BALF; **(B)** BALF levels of proinflammatory cytokines; The data are expressed as means ± SD. **(C)** Representative H&E staining of lung tissues, bars, 100 μm; **(D)** WT and Terc ^-/-^ mice (n = 8) were intratracheally (i.t.) challenged with VSV (1 x 10^8^ pfu/mice). Animals were monitored every 10 h for survival rate. The Kaplan-Meier and log-rank methods were used to analyze the data.

**
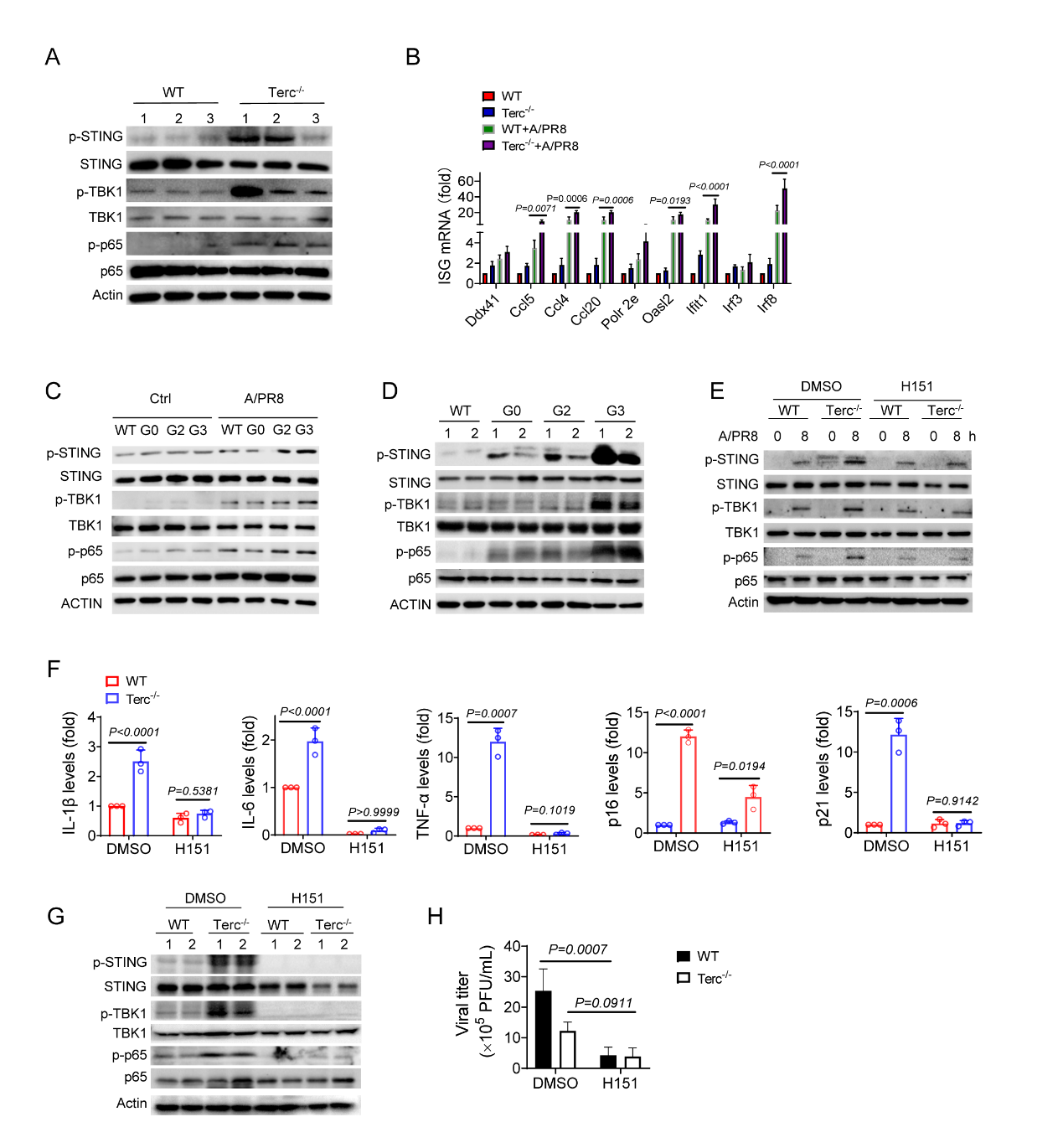
**

**Fig S3. Augmented STING activation in Terc^-/-^ macrophages but repressed by H151.** **(A, B)** WT and Terc^-/-^ mice (n=3) were treated as described in Figure 1B. Immunoblotting of STING and the downstream signaling molecules in BALF macrophages (A). qPCR quantification of lung expression of interferon-stimulatory genes (ISGs) (B). **(C, D)** Immunoblotting of STING and the downstream signaling molecules in WT, Terc^+/-^ (G0), second (G2) and 3^rd^ (G3) generation of Terc^-/-^ macrophages (C) or (D) mice (n=3) following A/PR8 infection. **(E)** Immunoblotting of STING and the downstream signaling molecules in WT and Terc^-/-^ macrophages pretreated with H151 or DMSO followed by A/PR8 stimulation for 8 h. **(F-H)** WT and Terc^-/-^ mice (n=3) were pretreated with H151 or DMSO and then subjected to A/PR8 infection. qPCR quantification of SASP molecules (F), and immunoblotting of STING and the downstream signaling molecules (G) in lungs; The relative viral loads in BALF (H). Shown are representative images and the data are expressed as means ± SD.


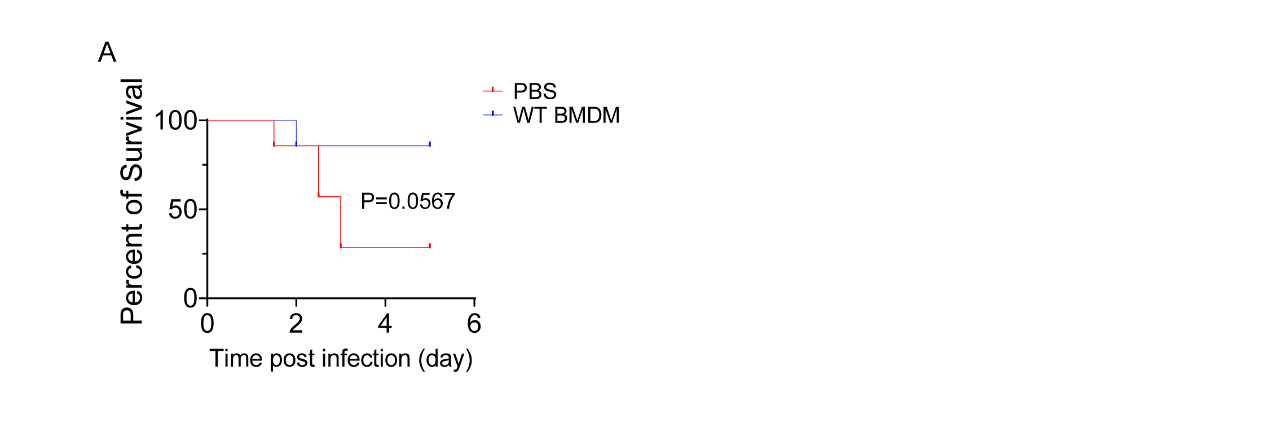


**Fig S4. Adoptive transfer of WT macrophages rescued the survival of infected Terc^-/-^ mice. (A**) Terc-/- mice (n=7) were intratracheally instilled with 1×108 PFU/mouse of A/PR8. BMDMs (2x106 / mouse) or PBS were subsequently administered intravenously into Terc-/- mice at -2, 1 and 3 d post infection. Animals were monitored every 10 h for survival rate. The Kaplan-Meier and log-rank methods were used to analyze the data.

**
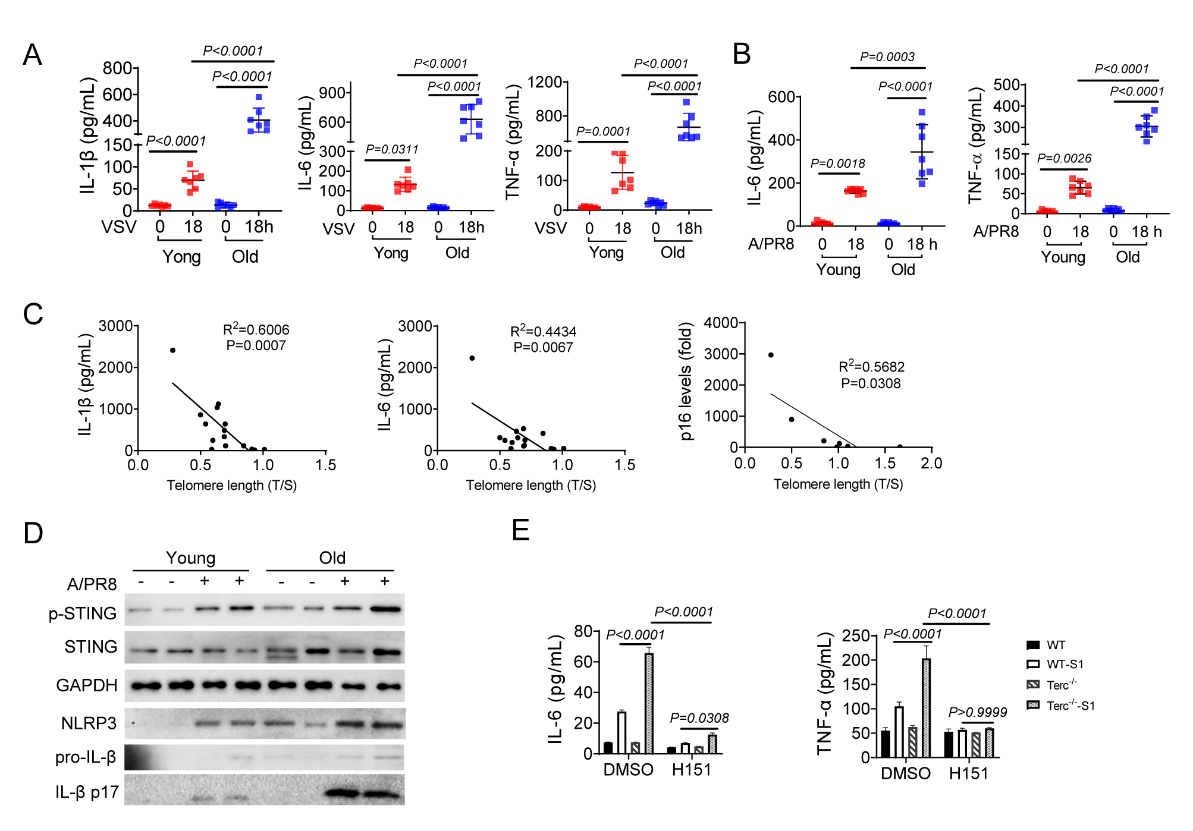
**

**Fig S5. Enhanced expression of proinflammatory cytokines by PBMCs from the aged upon viral infection. (A, B)** ELISA analysis of proinflammatory cytokines by PBMCs from young (<30 y) or old (>60 y) healthy subjects upon infection of VSV (A) or A/PR8 (B) for 18 h. **(C)** Reverse correlation between telomere lengths and the levels of IL-1β, IL-6, and p16. **(D)** Immunoblotting of the STING and NLRP3-related molecules in PBMCs from young and old healthy subjects. **(E)** ELISA analysis of IL6 and TNFα in macrophages pretreated with H151 or DMSO followed by stimulation with SARS-CoV-2 S1 for 18 h. Shown are representative images and the data are expressed as means ± SD.

**
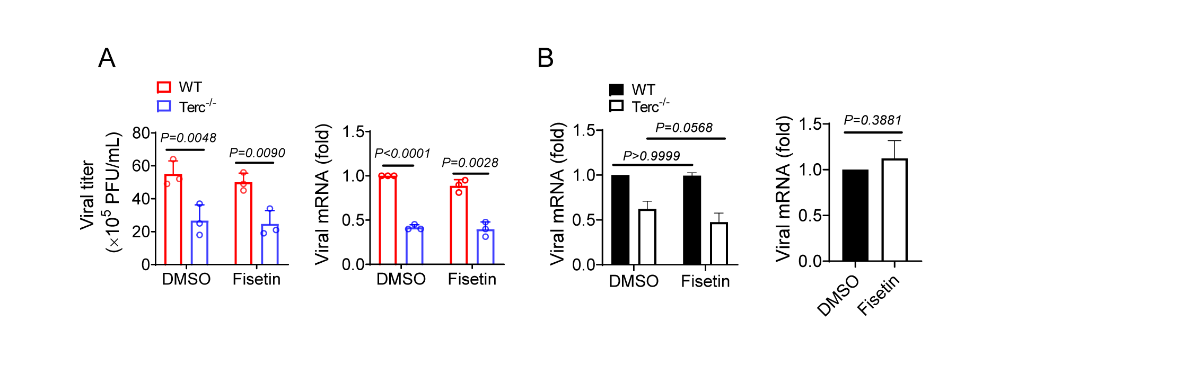
**

**Fig S6. Fisetin treatment does not affect viral loads.** WT and Terc^-/-^ mice were treated as described in Fig. 7A. **(A)** The relative viral loads were quantified in BALF and lung tissues. **(B)** Quantification of viral loads in BMDMs and A549 pretreated with fisetin or DMSO followed by PR/8 infection. Data are expressed as means ± SD.


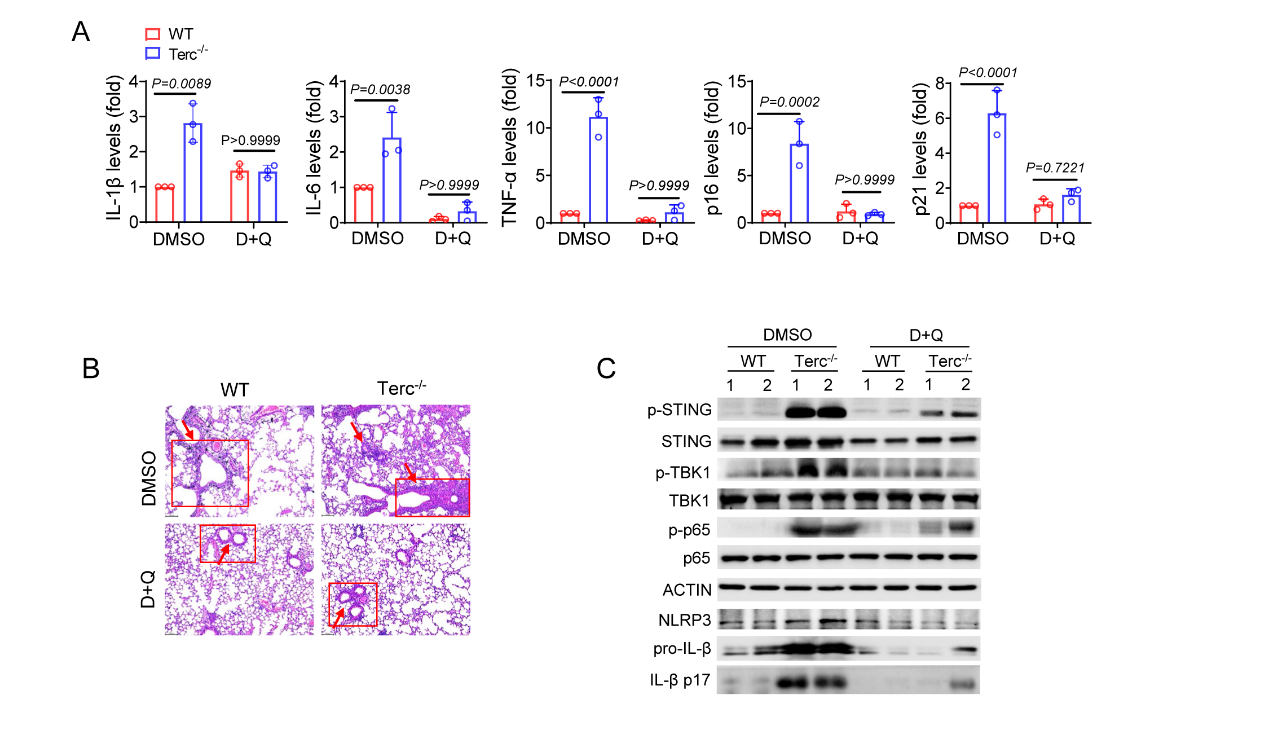


**Fig S7. D+Q treatment protects Terc^-/-^ mice from viral pneumonia.**

WT and Terc ^-/-^ mice (n = 3) were pretreated with D+Q (5 mg/kg +50 mg/kg) or DMSO for 5 days, followed by A/PR8 infection for 48 h. **(A)** qPCR quantification of lung SASP molecules. **(B)** Representative H&E staining of lung tissues, bars, 100 μm. **(C)** Immunoblotting of the STING/NLRP3 signaling molecules. Data are expressed as means ± SD.

**METHODS AND MATERIALS**

***Reagents***

The antibodies Phospho-TBK1/NAK (Ser172) (5483S), Phospho-NF-κB p65 (Ser536) (13346S), NF-Κb (8242S), NLRP3 (15101S), Cleaved Caspase-1 (67314S), Caspase-1 (3866S), IL-1β (12242S), Cleaved-IL-1β (63124S), ASC (67824), SIRT1 (8469) were from Cell Signaling; CDKN2A/p16INK4a (ab54210), p21 (ab188224), DRAQ5™ (ab108410) were from Abcam. STING (19851-1-AP), PGC1a (66369-1-Ig), COX15 (11441-1-AP), NDUFV2 (15301-1-AP), ATP5H (17589-1-AP), ATP5D (14893-1-AP) were from Proteintech, TBK1 (sc-398366) was from Santa Cruz. Phospho-STING (Ser366) was from Affinity. MitoTracker Green (M7514), Mito Tracker Red (M22425), and Mito SOX (M36008) were from Invitrogen.

***Mice***

Telomerase RNA component Terc knockout (Terc-/-) mice were gifted from Professor K.L. Rudolph and maintained in SPF environment. Terc-/- mice used in experiments were the third-generation offspring of homozygous Terc-/- mice. All animal experiments were performed in accordance with the National Institutes of Health Guide for the Care and Use of Laboratory Animals, and approved by the Animal Care and Use Committee of Nanjing University of Chinese Medicine.

***Cells and viruses***

Mice peritoneal cavity macrophages and Madin-Darby Canine Kidney (MDCK) cells were grown in complete DMEM medium. H1N1 A/Puerto Rico/8/1934 strain of influenza virus was propagated and titrated in MDCK cells. MDCK monolayer cells were inoculated with a 10-fold serial dilution of virus sample for 12 h at 37°C, then the cytopathic effect (CPE) was recorded and the TCID 50 was calculated using Reed-Muench method. TCID 50 value was converted into plaque-forming units (PFU) for further administration. Viruses were aliquoted and stored at -80°C.

***Quantitative PCR***

Total RNA was isolated using TRIzol reagent (Invitrogen) according to manufacturer’s instructions. First-strand cDNA was synthesized using PrimerScript II 1st Stand cDNA Synthesis Kit (Vazyme). Quantitative real-time PCR was performed using SYBR Green PCR Master Mix (YEASEN) to detect mRNA levels of target genes. The data were determined by the ΔΔCt method. Primers used in this study were synthesized by Generay (Nanjing, China) and corresponding sequences are listed in table s1.

***Determination of Cytokine Levels***

The levels of TNFα, IL-6 and IL-1β in the cell culture supernatants and BALF were measured by ELISA kits (R&D Systems) according to manufacturers’ instructions.

***Immunoblot***

Cell lysates were prepared by RIPA containing protease inhibitor cocktail and phosphatase inhibitor cocktail (YEASEN). Equal amounts of total protein were separated on SDS-polyacrylamide mini-gels and transferred onto Immobilon PVDF membranes (Millipore). After blocked in 5% Skim Milk (BD), membranes were incubated with appropriate antibodies overnight, followed by the incubation of secondary antibody conjugated with horseradish peroxidase. The immunoblotted proteins were visualized with an ECL detection reagent (Yeasen).

***ASC Oligomerization***

Briefly, after IAV infection, BMDMs were washed in PBS and lysed in 500 μL ice-cold buffer (50 mM Tris-HCl pH 7.6, 0.5% Triton X-100, 0.1 mM PMSF, and a protease inhibitor cocktail). The lysate was centrifuged at 330 g for 10 min and the pellets were washed and resuspended in PBS. Disuccinimidyl suberate (2 mM) was added to the resuspended pellets and followed by incubating at room temperature for 30 min with rotation. Samples were then centrifuged and re-suspended in the SDS loading buffer for western blotting.(Chi, et al. 2020)

***Histologic analysis of Lung Tissues***

The lung samples were washed thoroughly in PBS, fixed in 4% PFA; 5 μM sections were then stained with hematoxylin and eosin (H&E) using standard procedures and analyzed with Leica DMi1 Inverted microscope equipped with a Leica MC170HD CCD.

***Telomere length by quantitative PCR (qPCR)***

Total DNA was extracted from PBMCs using FastPure® Blood/Cell/Tissue/Bacteria DNA Isolation Mini Kit (Vazyme). The primer sequences ( 5′→3′) were:

tel F (300 nM), GGTTTTTGAGGGTGAGGGTGAGGGTGAGGGTGAGGGT;

tel R (300 nM), TCCCGACTATCCCTATCCCTATCCCTATCCCTATCCCTA;

36B4 F (300 nM), CAGCAAGTGGGAAGGTGTAATCC;

36B4 R (500 nM), CCCATTCTATCATCAACGGGTACAA.

All PCRs were performed on ABI QuantStudio 3 and the real-time PCR cycler as follow: 5 min at 95 ℃, 25 cycles (telomere reaction) and 35 cycles (single gene reaction) of 7 sec at 98 ℃ and 10 sec at 60 ℃ (telomere) or 10 sec at 58 ℃ (single gene). The final terminal length is the ratio of T (telomere)/S (single gene).(Gutierrez-Rodrigues, et al. 2014)

| **Table S1: the primers of RT-PCR** | | |
| --- | --- | --- |
| Gene | Forward primer (5′–3′) | Reverse primer (5′–3′) |
| Il1β | TGCCACCTTTTGACAGTGATG | TGATGTGCTGCTGCGAGATT |
| Il6 | GTCCTTCCTACCCCAATTTCCA | TAACGCACTAGGTTTGCCGA |
| Tnfα | CCTCACACTCACAAACCACCA | ACAAGGTACAACCCATCGGC |
| Ndufa4 | CTGGAGCAGCACTGTATGTGA | TTGGGACCCAGTTTGTTCCAT |
| Ndufa8 | GAGTTTATGCTGTGCCGCTG | TACTCTGTGAAAGGCTCCGC |
| Ndufa11 | TCCGCTTACAGCGTCTCAC | AGGCCAAACATCGCTCCAAT |
| Ndufb9 | ACCGGTACTTTGCTTGCTTG | ATCTCTCGAAGGAAGTGCCC |
| Ndufv1 | TGCTTGTGGCTCCGACTATG | ACAGTTGTGGGGCATCCAAA |
| Sdhb | CAGAGTCGGCCTGCAGTTT | ATCCAACACCATAGGTCCGC |
| Sdhd | CTGGTTCCAAGGCTGCATCT | AGCCAGAGAGTAGTCCACCA |
| Cyc1 | ATCGTTCGAGCTAGGCATGG | GCCGGGAAAGTAAGGGTTGA |
| Uqcr11 | GGAACTGGCCAGAAACTGGA | TGCCGTTGATGTAAGGCACC |
| Uqcrc1 | ATGCTGCGTGACATTTGCTC | TAGAAGCGCAGCCAGAACAT |
| Cox6a1 | CAACGTGTTCCTCAAGTCGC | CTTCATAGCCGGTCGGAAGT |
| Cox6b1 | AGAACTACAAAACTGCCCCCT | TTCTCACAGCGGTGGAAGTC |
| Cox5a | TGTCTGTTCCATTCGCTGCT | AACCGTCTACATGCTCGCAA |
| Cox5b | GCTTCAAGGTTACTTCGCGG | ATGGGTCCAGTCCCTTCTGT |
| Cox7c | GAGTATCCGGAGGTTCACGAC | ACCGCCACTTGTTTTCCACT |
| Cox8a | CAGGTCCACTCGAAGCCG | CAGGCAGAAGACAACACACG |
| Cox15 | GCGTCCGGCAACGGT | TGATGGTGCTGTACTGTCCT |
| Atp5d | TACGCTGACTGGAGCCTTTG | GTCCAGCATGTCCAGTGTCA |
| Atp5g1 | GCCAAGTTCATTGGTGCTGG | GGAGAAGAGCTGCTGCTTGA |
| Atp5g2 | ATGTACGCCTGCTCCAAGTT | CTGTGGTCGCTTCAACTCCA |
| Atp5g3 | CAGCTGATCCGAAGGGAGTTT | TGAAGGGTTTCAGCACCAGAA |
| Atp5h | TGGAATGAGACCTTCCACGC | GCACAGGAATCTTCAGGGCA |
| Atp5j2 | GAGCTGCCGAGCTGGATAAT | GGACCATGCTAATCCCCGAG |
| Atp5k | GGTTCAGGTCTCTCCACTCA | CTCCGCTGCTATTCTCCTCTC |
| Atp5e | TCAGCTACATCCGGTTTTCCC | TTTTATGCTGCTGCCCGAAG |
| Gene | Forward primer (5′–3′) | Reverse primer (5′–3′) |
| Atp6v1 | ACATCGCAGAGATGGTTCGG | CTTTGGCTGCATCGTAGGGA |
| Atp6v0c  Ifnβ  Pr8 | CATCGTCGGAGATGCTGGTG  CAGTAATAGCTCTTCAAGTGG  CCGAAGCTTTCTGGAAAATGATCT | GGAGAGGATTAGGGCCACGA  AGACTATTGTTGTACGTCTCC  GTCTCGAGTTACTCTAGCTCTA TGC |
| Pai1  Pai2  P16  P21  Oasl2  Ifit1  Irf3  Irf8  Ddx41  Ccl5  Ccl4  Ccl20  Polr2e | TCTGGGAAAGGGTTCACTTTACC  GTGCTGGGGGTAACACTGAAC  AAAGCGAACTCGAGGAGAGC  GTGGTGGAGACCTGATGATACC  TACCCACAGAGAGGGCAAAAG  ATCGCGTAGACAAAGCTCTTC  GAGAGCCGAACGAGGTTCAG  CGGGGCTGATCTGGGAAAAT  AGTCCGCCAAGGAAAAGCAA  GCTGCTTTGCCTACCTCTCC  TTCCTGCTGTTTCTCTTACACCT  GCCTCTCGTACATACAGACGC  GGTGGGCATCAAGACCATCAA | GACACGCCATAGGGAGAGAAG  GCGAAATCACAGCCACTGAAG  CGTGAACGTTGCCCATCATC  TATTCTGCTGGCAAAGTGGGA  GCATCGTAAGCTGGGAGGATA  GTTTCGGGATGTCCTCAGTTG  CTTCCAGGTTGACACGTCCG  CACAGCGTAACCTCGTCTTC  CTCAGACATGCTCAGGACATAAC  TCGAGTGACAAACACGACTGC  CTGTCTGCCTCTTTTGGTCAG  CCAGTTCTGCTTTGGATCAGC  TCAGGGACTAGCTCGTGCTC |
| Dloop1 | TGAACGGCTAAACGAGGGTC | AGCTCCATAGGGTCTTCTCGT |
| Dloop2 | CAGTCCCCTCCCTAGGACTT | ACCCTGGTCGGTTTGATGTT |
| Dloop3 | TAATCGCACATGGCCTCACA | GAAGTCCTCGGGCCATGATT |
| gDNA B2m | AGCAAAGAGGCCTAATTGAAGTC | GAAGTAGCCACAGGGTTGGG |

**Table S2: Clinical sample information**

| Patient | Age | Gender |
| --- | --- | --- |
| A1 | female | 24 |
| A2 | female | 23 |
| A3 | female | 22 |
| A4 | female | 26 |
| A5 | female | 28 |
| A6 | female | 22 |
| B1 | male | 29 |
| B2 | male | 27 |
| B3 | male | 22 |
| B4 | male | 26 |
| B5 | male | 21 |
| B6 | male | 26 |
| B7 | male | 25 |
| B8 | male | 28 |
| C1 | female | 60 |
| C2 | female | 81 |
| C3 | female | 63 |
| C4 | female | 61 |
| C5 | female | 66 |
| D1 | male | 65 |
| D2 | male | 64 |
| D3 | male | 69 |
| D4 | male | 69 |
| D5 | male | 65 |
| D6 | male | 60 |
| D7 | male | 68 |
| D8 | male | 74 |
| D9 | male | 74 |

**References**

Chi, Z., et al.

2020 Histone Deacetylase 3 Couples Mitochondria to Drive IL-1beta-Dependent Inflammation by Configuring Fatty Acid Oxidation. Mol Cell 80(1):43-58 e7.

Gutierrez-Rodrigues, F., et al.

2014 Direct comparison of flow-FISH and qPCR as diagnostic tests for telomere length measurement in humans. PLoS One 9(11):e113747.
